# Angiogenic and molecular diversity determine hepatic melanoma metastasis and response to anti-angiogenic treatment

**DOI:** 10.1186/s12967-022-03255-4

**Published:** 2022-02-02

**Authors:** Sebastian A. Wohlfeil, Verena Häfele, Bianca Dietsch, Céline Weller, Carsten Sticht, Anna Sophia Jauch, Manuel Winkler, Christian David Schmid, Anna Lena Irkens, Ana Olsavszky, Kai Schledzewski, Philipp-Sebastian Reiners-Koch, Sergij Goerdt, Cyrill Géraud

**Affiliations:** 1 Department of Dermatology, Venereology, and Allergology, University Medical Center and Medical Faculty Mannheim, Heidelberg University, and Center of Excellence in Dermatology, 68135 Mannheim, Germany; 2 Section of Clinical and Molecular Dermatology, Medical Faculty Mannheim, Heidelberg University, Mannheim, Germany; 3 European Center for Angioscience, Medical Faculty Mannheim, Heidelberg University, Mannheim, Germany; 4 NGS Core Facility, Medical Faculty Mannheim, Heidelberg University, Mannheim, Germany

**Keywords:** Cutaneous melanoma, Melanoma metastasis, Liver metastasis, Tumor heterogeneity, Anti-angiogenesis, Sorafenib

## Abstract

**Background:**

Cutaneous melanoma exhibits heterogeneous metastatic patterns and prognosis. In this regard, liver metastasis, which is detected in ~ 10–20% of stage 4 patients, came to the fore of melanoma research, as it recently evolved as decisive indicator of treatment resistance to immune checkpoint inhibition.

**Methods:**

Hepatic metastases were induced by intrasplenic injection of five different murine melanoma cell lines. The efficiencies of hepatic colonization, morphologic patterns, gene expression profiles and degree of vascularization were analyzed and Sorafenib was applied as anti-angiogenic treatment.

**Results:**

WT31 melanoma showed the highest efficiency of hepatic colonization, while intermediate efficiencies were observed for B16F10 and RET, and low efficiencies for D4M and HCmel12. RNAseq-based gene expression profiles of high and intermediate metastatic melanomas in comparison to low metastatic melanomas indicated that this efficiency predominantly associates with gene clusters involved in cell migration and angiogenesis. Indeed, heterogeneous vascularization patterns were found in the five models. Although the degree of vascularization of WT31 and B16F10 metastases differed, both showed a strong response to Sorafenib with a successful abrogation of the vascularization.

**Conclusion:**

Our data indicate that molecular heterogeneity of melanomas can be associated with phenotypic and prognostic features of hepatic metastasis paving the way for organ-specific anti-angiogenic therapeutic approaches.

**Supplementary Information:**

The online version contains supplementary material available at 10.1186/s12967-022-03255-4.

## Background

Cutaneous melanoma (CM) is a phenotypic and molecular heterogeneous disease arising from melanocytes of the skin and preferentially spreads to the skin, lymph nodes, lung, liver and brain [1]. In recent years, the treatment of metastatic CM has been revolutionized by immune checkpoint inhibition (ICI) and targeted therapies (BRAF/ MEK inhibitors (BRAFi/MEKi)). Meanwhile, a median survival of 60 months is achieved by combined ICI [2]. And even for BRAF-mutated melanoma treated with combined BRAFi/MEKi durable treatment responses are seen in subpopulations with favorable prognostic factors such as normal or low LDH and low volume of disease [3]. Despite this huge progress in melanoma therapy, liver metastasis of CM is well documented as predictor of poor response to ICI [4] or targeted therapy of BRAF-mutated melanoma [5]. Therefore, it is important to develop novel treatment options for melanoma patients with advanced disease suffering from liver metastases.

Recent studies have provided insight into mechanisms of global therapy resistance mediated by hepatic metastasis. In murine models of colorectal carcinoma (CRC) and melanoma, subcapsular injection of MC38 CRC or B16F10 melanoma cell lines into the liver abolished the response of corresponding subcutaneous tumors to ICI. The authors tie this to infiltration of regulatory T-cells into the subcutaneous tumors which in turn recruit CD11b^+^ monocytes [6]. Most recently, these findings were extended by the fact that liver metastases of CRC and melanoma recruit tumor-specific CD8^+^ T-cells from the periphery, which underwent apoptosis, and, as consequence, induced systemic immunosuppression and reduced response to ICI [7].

Current clinical research focuses on sequential and combined treatment approaches of ICI and targeted therapies [8]. Moreover, additional compounds targeting components of the tumor microenvironment such as macrophages, fibroblasts or blood vessels are tested in preclinical studies to improve therapeutic options for melanoma patients suffering from liver metastasis.

In general, liver metastases most commonly arise from CRC, pancreatic, lung or mammary carcinoma. Regarding CRC or pancreatic cancer, the liver is the first organ in line of the vascular tree and therefore could be considered as sieve for disseminating tumor cells. In contrast, this does not apply for lung or mammary carcinoma, uveal melanoma (UM) or CM. Paget postulated that both tumor-intrinsic factors (“seeds”) as well as the microenvironment of the target organ (“soil”) need to be taken into account to understand organotropic metastasis [9]. UM is therefore often seen as paragon of this hypothesis, as it shows a remarkable proclivity for liver colonization as in nearly 90% of metastatic patients hepatic lesions are detected [10].

Research has often focused on phenotypic characteristics of liver metastasis in these tumor entities. Morphologic characterization identified pushing, replacement and desmoplastic histological growth patterns (HGPs) of breast cancer, colorectal, UM and CM liver metastases [11–13]. In CM almost 55% of liver metastases are pure desmoplastic and associated with an improved prognosis as compared to any replacement HGP. The pushing HGP occurs in around 12% of cases and leads to rapid mortality [13]. Interestingly, the desmoplastic HGP of CRC correlates with an increased response to anti-angiogenic therapy and strong immune cell infiltration [14, 15].

In regard to tumor cell-intrinsic mechanisms regulating organotropic liver metastasis epithelial-to-mesenchymal transition controlled by miR-200c and the PKCζ/ADAR2 axis is a decisive step to hepatic metastasis of CRC [16]. Moreover, SMAD3 mutations in CRC liver metastasis are associated with the poorest prognosis [16–18]. In breast cancer, human epidermal growth factor receptor 2 (HER2)-enriched subtypes significantly correlate with increased hepatic metastasis [19, 20]. At the molecular level reduced BMP-SMAD1/5 signaling is related to decreased distant metastasis free and decreased overall survival of patients [21]. This pathway controls breast cancer metastasis in an organ-specific manner as pharmacological inhibition by tacrolimus with or without addition of a MEK inhibitor reduces metastasis to liver and bone while lung and brain metastasis are not affected. In UM, loss of the tumor suppressor BRCA1-associated protein 1 (BAP1) is the key event to metastasis and correlates with disease outcome [22, 23].

In CM, mutations of both NRAS and BRAF correlate with brain and liver metastasis [24]. However, this correlation is weak as NRAS or BRAF mutations are found in approximately 80% of melanomas and cannot be used as reliable clinical predictor of hepatic metastasis. So far, no mutational drivers of organ-specific hepatic metastasis have been described for CM. However, a liver passaged B16 melanoma subline shows increased expression of integrin alpha2 and enhanced liver but not lung metastasis [25]. Besides, tumor-intrinsic MSX1 expression regulating neural crest-like reprogramming is associated with preferential metastasis to the liver. Yet it is not known whether this also occurs in an immunocompetent setting as this study was performed in immunodeficient mice only [26].

Tumor intrinsic features associated with hepatic colonization of CM have not been characterized in detail. Therefore, this study comparatively analyzed five genetically different murine melanoma cell lines, reflecting the genetic heterogeneity of melanomas, and their molecular and phenotypic features underlying the different efficiencies of liver metastasis formation in mice.

## Methods

### Animals

For in vivo experiments female C57Bl/6 wildtype mice were purchased by Janvier. For metastasis experiments mice were age-matched and were used at least at 10 weeks of age. All animals were hosted in single ventilated cages (Sealsafe plus DGM™, Techniplast, Italy; Bedding H0234-20, Ssniff, Germany) in a 12 h/12 h day/night cycle under Specific-pathogen-free conditions and fed ad libitum with a standard rodent diet (ssniff®R/M-H autoclavable, V1534-000, Ssniff, Germany).

### Cell lines

All used melanoma cell lines were from murine origin. The melanoma cell line B16F10 *luc2* was purchased from Perkin Elmer (MA, USA). RET1 melanoma cells were generated from metallothionein-I (MT)/*RET* transgenic mice [27] and kindly provided by V. Umansky (German Cancer Research Center (DKFZ), Heidelberg, Germany). The transformed melanoma cell line WT31 was derived from *Tyr*∷*Nras*^*Q61K/*°^*; INK4a*^*−/−*^ mice [28] and was a generous gift from O. Sansom (Beatson Institute for Cancer Research, Scotland). HCmel12 melanoma cells were established from a primary melanoma in HGF-CDK4(R24C) mice [29] and kindly provided by T. Tüting (University of Magdeburg, Germany). The Dartmouth murine mutant malignant melanoma (D4M) is derived from Tyr::CreER;Braf^CA^;Pten^lox/lox^ mice [30] and was generously provided by C. E. Brinckerhoff (Geisel School of Medicine at Dartmouth, NH, USA). For cell authentication STR sequencing was performed (Eurofins, Ebersberg, Germany) and confirmed unique profiles of all used cell lines. Besides, cells were distinguished by pigmentation status, morphology or bioluminescence. All cell lines were regularly tested mycoplasma-free by PCR. B16F10 *luc2*, WT31 and RET cells were maintained in RPMI 1640 media (Thermo Fisher Scientific, MA, USA) with 10% (v/v) fetal calf serum (FCS) and 100 U/ml penicillin/streptomycin at 37 °C, 5% CO_2_. HCmel12 cells were cultured in RPMI with 10% (v/v) FCS, Hepes, ß-Mercaptoethanol, 1% L-glutamine, 100 U/ml penicillin/streptomycin, 1% Natrium Pyruvate and 1% NEAA. D4M cells were maintained in Advanced DMEM/F-12 (Thermo Fisher Scientific, MA, USA) with 5% (v/v) FCS, 100 U/ml penicillin/streptomycin and glutamine. For in vivo experiments always the same passage of corresponding cell lines was used. After thawing they were not passaged more than three times and maximum culture time prior to in vivo experiments was one week.

### Liver colonization assays

Spleen injection of melanoma cells was performed as described previously [31]. Corresponding cell numbers of B16F10 *luc2*, RET, D4M or HCmel12 ranged from 0.5 × 10^5^ to 3.0 × 10^5^, please refer to individual Figure legends. Regarding WT31 melanoma cells, 0.1 × 10^5^ or 0.3 × 10^5^ were used for spleen injection. For i.v. injection, 1.25 × 10^6^ to 2.5 × 10^6^ WT31 cells were injected. The animals were sacrificed at indicated time points, at least at day 21. Organs were removed and analyzed for melanoma colonization.

### Application of Sorafenib

Mice were injected with 0.3 × 10^5^ WT31 melanoma cells intrasplenically. From day 1 to 18 mice received daily i.p. injections of Sorafenib (Sigma-Aldrich, MS, USA) (60 mg/kg KG, diluted in 12.5% Cremophor (Sigma-Aldrich, MS, USA), 12.5% ethanol, and 75% sterile saline) or vehicle/solvent control (12.5% Cremophor, 12.5% ethanol, and 75% sterile saline). At day 19 mice were sacrificed and metastases were quantified.

### Liver dissection, cryopreservation, and paraffin embedding

Mice were sacrificed by cervical dislocation. Livers were fixed in 4% PFA at 4 °C for 24 h, followed by paraffin embedding according to standard protocols. As well, livers were embedded in OCT (Sakura Finetek Europe B.V. KvK, Netherlands).

### Immunohistochemistry and immunoflurescence

Deparaffinization and rehydration of paraffin sections (1–5 µm) was performed according to standard protocols. Antigen retrieval was carried out with epitope retrieval solution (Zytomed Systems, Germany) at either pH 6, pH 8 or pH 9. Cryosections (8 µm) were fixed with 4% PFA or re-hydrated in PBS when PFA perfused, pre-fixed livers were cut. First antibody was incubated over night at 4 °C, secondary antibody was applied for 1 h at room temperature after three washing steps with PBS. Sections were mounted with Dako fluorescent mounting medium (Dako, Agilent technologies, USA). Staining of paraffin sections was performed as previously described [32]. For hematoxylin & eosin (H&E), periodic acid-Schiff (PAS), Prussian blue and Sirius red staining, formalin-fixed, paraffin-embedded samples were processed according to standard protocols provided by the manufacturer.

### Image acquisition and processing

Pictures of routine histology stainings were acquired by Nikon Eclipse Ni-E (Nikon Instruments Europe BV, Amsterdam, Netherlands) using a 10x/0.45 plan apochromat objective. During acquisition data were not compressed. Pictures were processed in NIS-Elements (Nikon Instruments Europe BV, Amsterdam, Netherlands).

### Confocal microscopy

Analysis of fluorescent-labelled sections was performed with a TCS SP5 DS laser scanning spectral confocal microscope (Leica Microsystems, Germany). For excitation wavelengths were set to 488, 543 and 633 nm. To visualize Alexa Fluor 488, Cy3 and Alexa Fluor 647 conjugates the emission maxima were detected at 518, 570 and 673 nm. Three representative areas per samples were chosen and images were acquired in a sequential mode. Processing of images was performed by Leica confocal software (Leica Microsystems, Germany) and ImageJ software (NIH, USA). In detail, color thresholds were set in relation to the whole image (= fluorescent area). Endothelial marker expression was quantified using Image J. First, the metastatic area was marked manually and auto thresholding was applied. Then the lower threshold was adjusted to represent the positive signal. Afterwards, the areas positive for marker expression inside and outside the metastatic area were calculated and measured as area fraction. Last, the percentage of a certain marker expression was set in relation to the total intratumoral vessel area.

### Antibodies

Primary antibodies: Rabbit anti-cleaved Caspase 3 (9661S, Cell Signaling Technology, USA), rabbit anti-Ki-67 (ab16667, Abcam, UK), rat anti-CD31 (DM3614P, Dianova, Germany), goat anti-CD32b (AF1460, R&D Systems, USA), rabbit anti-Stabilin-2 peptide 15 antibody [33], goat anti-Lyve1 (AF2125, R&D Systems, USA), rat anti-Endomucin (14–5851-82, Thermo Fisher Scientific, USA), rabbit anti-TRP-2 (ab74073, Abcam, UK), goat anti-Lama4 (AF3837, R&D Systems, USA), rabbit anti-Desmin (ab15200, Abcam, UK), rabbit anti-Fibronectin (ab23750, Abcam, UK), rabbit anti-Collagen I (R1038, Acris Antibodies, Germany), rabbit anti-Collagen III (R1040, Acris Antibodies, Germany), rabbit anti-Collagen IV (NB120-6586, Novus Biologicals, Germany). Secondary antibodies: Alexa-Fluor 488, Alexa-Fluor 647 and Cy3-conjugated secondary antibodies were purchased from Dianova (Germany).

### RNA isolation and RNA sequencing

Total RNA from sub-confluent (50%), cultured melanoma cells was extracted with innuPREP RNA Mini Kit (845-KS-2080250, Analytik Jena, Germany), then treated with TURBO DNA-free Kit (AM1907, Invitrogen, USA). Samples were prepared and RNA concentration and quality were measured by a NanoPhotometer NP80 (Implen, Munich, Germany) and a 2100 Bioanalyzer (Agilent Technologies, Santa Clara, CA, USA). The library preparation and the sequencing with an Illumina HiSeq 4000 sequencing system (Illumina, CA, USA) were then performed by BGI (Hongkong, China). The raw and normalized gene expression profiling data have been deposited in NCBI's Gene Expression Omnibus and are accessible through GEO Series accession number GSE185539.

### RNA sequencing data analysis

Most of the procedure was done with R and Bioconductor using the NGS analysis package systempipeR [34]. The quality control of raw sequencing reads was performed using FastQC (Babraham Bioinformatics, UK). Low-quality reads were removed using trim_galore (version 0.6.4). The resulting reads were aligned to mouse genome version GRCm38.p6 from GeneCode and counted using kallisto version 0.46.1 [35]. The count data was transformed to *log2*-counts per million (logCPM) using the voom-function from the limma package [36]. Differential expression analysis was performed using the limma package in R. A false positive rate of α = 0.05 with FDR correction was taken as the level of significance. Volcano plots and heatmaps were created using ggplot2 package (version 2.2.1) and the complexHeatmap (version 2.0.0) [37]. Pathway analyses were made with fgsea package [38] and the enrichmentbrowser package [39] in R using the pathway information from KEGG database (URL: https://www.genome.jp/kegg/pathway.html). The GSEA was made with R.

### qPCR

We performed reverse transcription with Maxima Reverse Transcriptase (EP0752, Thermo Fisher Scientific) and Oligo(dT)18 primers (SO131, Thermo Fisher Scientific) according to the manufacturer’s instructions. innuMIX qPCR SyGreen Sensitive (845-AS-1310200, Analytik Jena, Jena, Germany) was used on a qTOWER 3 G touch thermal cycler (Analytik Jena) for quantitative PCR (qPCR) of cDNA. qPCR primers were designed with NCBIs PrimerBLAST (https://www.ncbi.nlm.nih.gov/tools/primer-blast/). For mRNA specificity, qPCR primers were designed to span an exon-exon junction where possible. Primers were tested with no template controls, original RNA, and melt curve analysis. Primer sequences are listed in Additional file 2: Table S4. qPCR output files were analyzed in qPCRsoft 4.0.8.0 (Analytik Jena) and normalized expressions (ddCt algorithm) were calculated using the reference genes Gak and Srp72.

### Statistical analysis

All statistical analyses and graphical displays were performed with GraphPad Prism7 (Graph Pad, USA) and mean ± SEM is presented. For statistical analysis, an unpaired, two-tailed *t*-test was applied if data met the criteria of normality. Otherwise, Mann–Whitney (U) test was used. For grouped analysis, a Dunn’s multiple comparisons test was applied when data were not distributed normally. For analysis of qPCRs a one-way ANOVA was performed followed by a correction for multiple comparisons by a Tukey test. Differences between data sets with P < 0.05 were considered statistically significant.

## Results

**Fig. 1:**
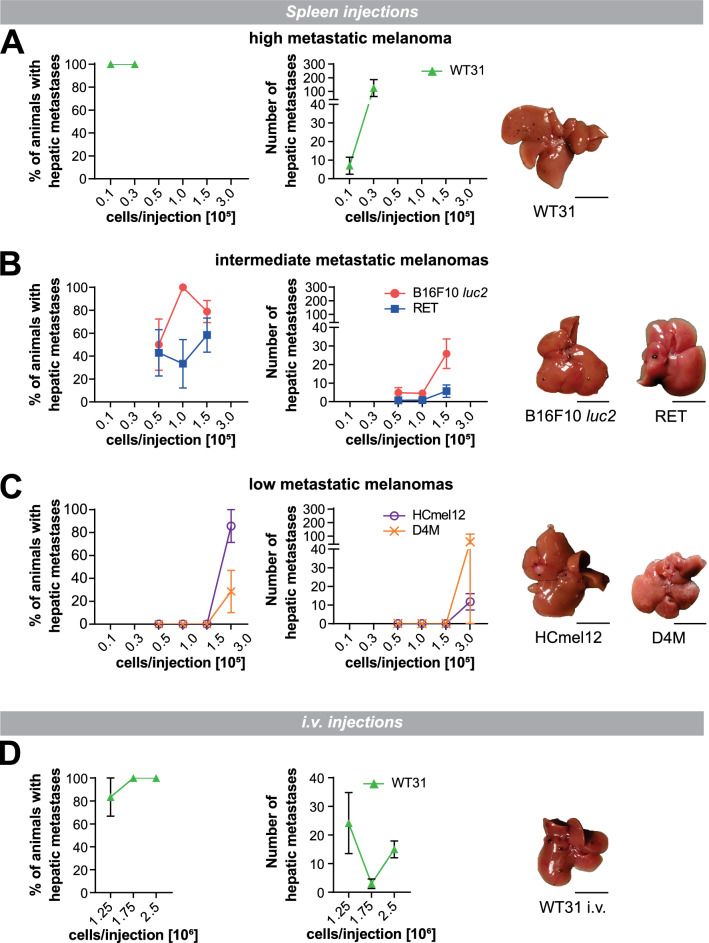
Melanoma cell lines differ in liver colonization efficiency and in the number of hepatic metastases. **A**–**C** WT31 (high metastatic), B16F10 *luc2*, RET (intermediate metastatic), D4M and HCmel12 (low metastatic) melanoma cells were injected intrasplenically at indicated cell numbers (0.1, 0.3 or 0.5, 1.0, 1.5 × 10^5^ cells). Only D4M and HCmel12 were injected with 3.0 × 10^5^ cells. Mice were sacrificed at day 14 (WT31, B16F10, RET, D4M, HCmel12) or at day 21 (RET, D4M, HCmel12). A pooled analysis of day 14 and day 21 is presented. Macroscopic visible liver metastases were counted. The percentage of mice with hepatic metastases (efficiency of hepatic colonization) and the number of macroscopic liver metastases are displayed. Representative images of colonized livers are shown. Scale bars = 1 cm. For detailed statistical analysis refer to Additional file 2: Table S1 and S2. **D.** WT31 melanoma cells were injected intravenously at indicated cell numbers (1.25, 1.75, 2.5 × 10^6^). Mice were sacrificed at day 19. Macroscopic visible liver metastases were quantified. The percentage of mice with hepatic metastases (efficiency of hepatic colonization) and the number of macroscopic liver metastases are shown. A representative image of a liver with WT31 melanoma metastases is presented. Scale bar = 1 cm. For detailed analysis refer to Additional file 2: Table S3

Since CM differ in underlying driver mutations as well as their clinical and morphologic features, five murine melanoma cell lines with heterogenous driver mutations were selected (Table 1) to evaluate their ability to colonize the liver. These cell lines showed a highly variable efficiency of liver metastasis after intrasplenic injection (Fig. 1A–C; Additional file 1: Figure S1A; Additional file 2: Table S1, S2A,B). WT31 melanoma reliably led to development of hepatic metastases and high numbers of metastatic nodules even at low cell concentrations. In comparison, B16F10 *luc2* and RET showed a medium colonization efficacy and lower numbers of liver metastases. D4M and HCmel12 melanoma cells formed liver metastases at the highest cell concentrations injected indicating the lowest efficiency of hepatic colonization. Macroscopically, most of the tested melanoma cell lines developed black or grey round tumor nodules, except for D4M, which formed white, pinpoint-like lesions. In cell culture, cell pellets of WT31 melanoma were the most pigmented ones (Additional file 1: Figure S1B). Due to the strong and reliable liver colonization efficiency of WT31 melanoma after spleen injection, WT31 cells were also injected intravenously (Fig. 1D; Additional file 1: Figure S1A; Additional file 2: Table S3A, B). Interestingly, WT31 cells not only managed to colonize the liver even after intravenous (i.v.) injection, but metastases were also detected in the lungs, the brain, the bones, the kidneys and the spleen (Table 2; Additional file 1: Figure S1C).

**Table 1:** Driver mutations of melanoma cell lines used

Cell line	Driver mutation
B16F10	Multiple mutations, 563 in expressed genes, including *Pten*, *Trp53*. None in *Braf*, *c-Kit*, *Kras* or *Nras* [60]
RET1	Overexpression of human *RET* proto-oncogene driven by the mouse metallothionein1 (MT) promotor [27, 43]
WT31	*Tyr∷Nras*^*Q61K/°*^*; INK4a*^*−/−*^ [28]
HCmel12	HGF-CDK4(R24C) [29]
D4M	Tyr::CreER;Braf^CA^;Pten^lox/lox^ (30)

**Table 2:** Extrahepatic metastasis of WT31 melanoma after intravenous injection of 2.5 × 10^6^ cells

Organ	Number of animals with metastases (percentage)
Lungs	7/7 (100%)
Kidneys	6/7 (85.7%)
Bones	2/7 (28.6%)
Adipose tissue	1/7 (14.3%)
Spleen	1/7 (14.3%)
Ovary	1/7 (14.3%)

**Fig. 2:**
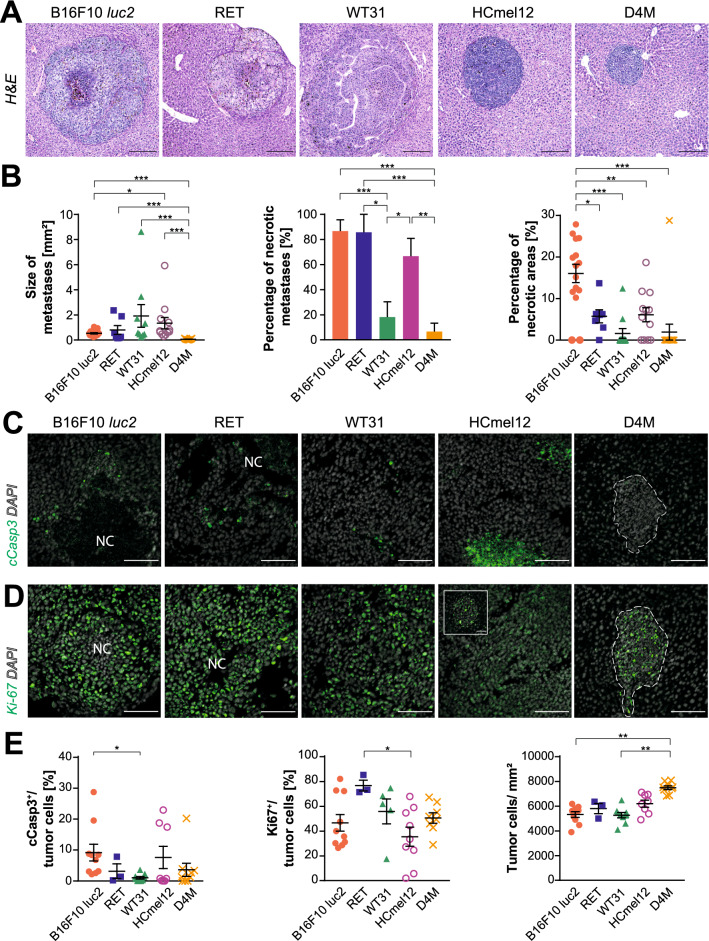
Melanoma liver metastases share a pushing type histopathological growth pattern, but differ in metastatic size, the number of necrosis and proliferation rate. **A.** Images of H&E stainings of hepatic metastases of B16F10 *luc2*, RET, WT31, HCmel12 and D4M melanoma. Scale bars = 200 µm, n = 5. **B.** The size of liver metastases was measured and area in mm^2^ is presented. A Dunn’s test was applied (p = 0.0338 for B16F10 vs. HCmel12; p < 0.0001 for B16F10 vs. D4M; p = 0.0004 for RET vs. D4M; p < 0.0001 for WT31 vs. D4M; p < 0.0001 for HCmel12 vs. D4M). Second, H&E stainings of liver metastases were analyzed for necrosis. The percentages of necrotic metastases in comparison to total number of metastases analyzed are presented. A Dunn’s test was applied (p = 0.0009 for B16F10 vs. WT31; p < 0.0001 for B16F10 vs. D4M; p = 0.0128 for RET vs. WT31; p = 0.0006 for RET vs. D4M; p = 0.0361 for WT31 vs. HCmel12; p = 0.0027 for HCmel12 vs. D4M). Third, the percentage of necrotic areas in comparison to total size of the metastases was quantified. A Dunn’s test was applied (p = 0.0107 for B16F10 vs. RET; p < 0.0001 for B16F10 vs. WT31; p = 0.0026 for B16F10 vs. HCmel12; p < 0.0001 for B16F10 vs. D4M). Number of animals analyzed = 5 (B16F10 *luc2*), 3 (RET), 5 (WT31), 6 (HCmel12), 4 (D4M). Number of metastases analyzed = 15 (B16F10 *luc2*), 7 (RET), 11 (WT31), 12 (HCmel12), 15 (D4M). **C.** Immunofluorescence stainings for cleaved Caspase3 (cCasp3) and DAPI. Representative images of the center of metastases are shown. Necrotic cores (NC) are marked. Scale bar = 100 µm, n = 5. Number of animals analyzed = 3 (B16F10 *luc2*), 3 (RET), 4 (WT31), 4 (HCmel12), 4 (D4M). Number of metastases analyzed = 10 (B16F10 *luc2*), 3 (RET), 9 (WT31), 8 (HCmel12), 9 (D4M). **D.** Immunofluorescence stainings for Ki-67 and DAPI. Representative images of the center of metastases are shown. Necrotic cores (NC) are marked. Scale bar = 100 µm, n = 5. Insert shows a representative picture of a smaller metastasis of HCmel12 melanoma. Scale bar = 100 µm. Number of animals analyzed = 3 (B16F10 *luc2*), 3 (RET), 3 (WT31), 4 (HCmel12), 5 (D4M). Number of metastases analyzed = 10 (B16F10 *luc2*), 3 (RET), 5 (WT31), 9 (HCmel12), 8 (D4M). **E.** The Percentages of cCasp3^+^ and DAPI^+^ (p = 0,0225 for B16F10 vs. WT31) or Ki-67^+^ and DAPI^+^ (p = 0,0360 for RET vs. HCmel12) as compared to total DAPI^+^ melanoma cells are shown. Dunn’s tests were performed. Besides, the number of DAPI^+^ tumor cells related to tumor area is presented (p = 0.0007 for B16F10 vs. D4M; p = 0.0002 for WT31 vs. D4M). A Dunn’s test was applied. Number of animals analyzed = 3 (B16F10 *luc2*), 3 (RET), 4 (WT31), 4 (HCmel12), 4 (D4M). Number of metastases analyzed = 10 (B16F10 *luc2*), 3 (RET), 9 (WT31), 8 (HCmel12), 9 (D4M). Data information: *P < 0.05, **P < 0.01, *** P < 0.0001, n.s. = not significant.

Histomorphologically, all melanoma metastases showed a pushing type HGP with no desmoplastic rim (Fig. 2A). Sirius Red and PAS stainings did neither reveal relevant fibrosis nor alterations of glycogen deposition (Additional file 1: Figure S2A, B). Histological measurements revealed that D4M formed the smallest metastases (0.06 mm^2^ ± 0.01) and WT31 the largest (1.92 mm^2^ ± 0.9) (Fig. 2B). The percentage of necrotic metastases strongly differed between the cell lines as D4M and WT31 showed the fewest necrotic cores (D4M: 6.7% ± 6.7; WT31: 18.2% ± 12.2), while B16F10 *luc2*, RET and HCmel12 presented significantly more necrotic areas (B16F10 *luc2*: 86.7% ± 9.1; RET: 85.7% ± 14.3; HCmel12: 66.7% ± 14.2) (Fig. 2B). Among the latter three, B16F10 *luc2* (16.1% ± 2.2) showed the largest necrotic areas as compared to RET (5.7% ± 1.6) and HCmel12 (6.1% ± 1.7) (Fig. 2B). Therefore, apoptosis and proliferation were assessed by cleaved Caspase3 (cCasp3) and Ki-67 staining. Here, the metastases formed by B16F10 *luc2* (9.2% ± 2.7) and HCmel12 (7.6% ± 3.6) revealed the most cCasp3-positive cells at the metastatic center (Fig. 2C, E). Besides, the highest proliferative index was found in RET melanoma metastases (76.7% ± 4.4) (Fig. 2D, E). Furthermore, tumor cell density was the highest in hepatic lesions of D4M (7493 cells per mm^2^ ± 147.5) indicating a smaller cell size (Fig. 2E).

**Fig. 3:**
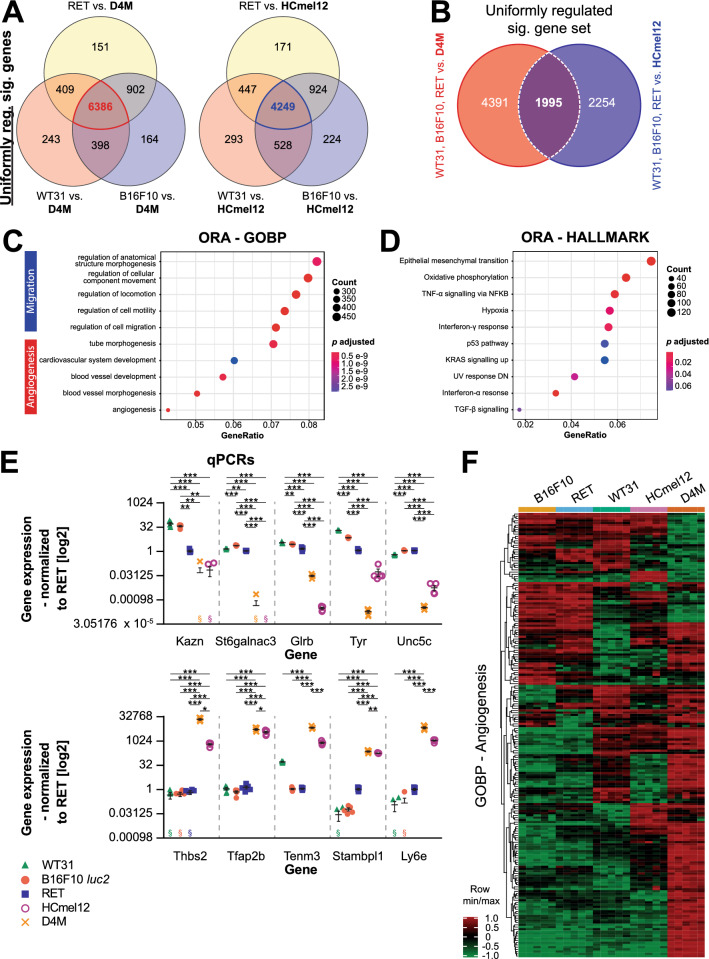
Differential gene set analysis of WT31, B16F10 luc2 and RET melanoma in comparison to D4M or HCmel12. RNA-seq of cultured, sub-confluent B16F10, RET, WT31, HCmel12 and D4M melanoma cells was performed for gene expression profiling. N = 5/ cell line. **A.** Uniformly regulated significant genes of B16F10 (blue), RET (yellow) or WT31 (red) in relation to D4M melanoma were compared by a Venn diagram. Besides, commonly regulated significant genes of B16F10 (blue), RET (yellow) or WT31 (red) in relation to HCmel12 melanoma were compared by a second Venn diagram. **B.** Identified gene sets with common regulation of significant genes of B16F10, RET and WT31 in relation to D4M (red) or HCmel12 (blue) were matched in a third Venn diagram. The overlay represents a gene set of 1995 significant genes which are uniformly regulated in B16F10, RET or WT31 as compared to D4M and HCmel12. **C, D.** The previously identified gene set was compared by an overall representation analysis of gene ontology biological processes (GOBP) (C) or HALLMARK pathways (D). Dot plots display both the number of regulated genes by count and gene ratio and the adjusted *p* value of corresponding pathways. **E.** Quantitative PCRs (qPCRs) of the ten genes with the strongest up- and downregulation. Relative expression was normalized to RET melanoma cells. Kazn, St6galnac3, Glrb, Tyr and Unc5c were the most downregulated genes in D4M and HCmel12 as compared to WT31, B16F10 *luc2* and RET. Thbs2, Tfap2b, Tenm3, Stambpl1 and Ly6e were the most upregulated genes in D4M and HCmel12 as compared to WT31, B16F10 *luc2* and RET. § = datapoint(s) with no detection of a ct value, corresponding dots could not be plotted due to the logarithmic scale. N = 5/ cell line. Detailed statistical analyses are provided in Additional file 2: Table S5. **F.** A heat map of the GOBP angiogenesis gene set is presented. Please refer to Additional file 1: Figure S5 for the complete heat map including gene symbols

To gain molecular and mechanistic insights into the pathways and processes influencing hepatic metastatic efficiency, all melanomas were analyzed by RNA-seq of cultured cells at the same confluency as used for in vivo experiments. High and intermediate metastatic melanoma cell lines (WT31, B16F10 *luc2* and RET) (HIM-melanoma) were therefore compared to the ones with low metastatic efficiency (D4M or HCmel12) (LM-melanoma). To select for commonly regulated genes and pathways, only significant genes with the same direction of regulation were considered among HIM-melanoma. First, uniformly regulated significant genes of HIM-melanoma were compared in relation to D4M identifying a gene set of 6386 regulated significant genes (Fig. 3A). Second, the same process was repeated with HCmel12 melanoma as reference identifying 4249 uniformly regulated significant genes (Fig. 3A). Last, these two gene sets were then matched to identify 1995 commonly regulated significant genes of HIM-melanoma in comparison to LM-melanoma (Fig. 3B). Subsequent pathway analyses of gene ontology biological processes (GOBP) (Fig. 3C) revealed significant regulation of processes involved in cell migration or angiogenesis. Moreover, gene enrichment analysis of HALLMARK pathways (Fig. 3D) demonstrated significant involvement of epithelial mesenchymal transition, oxidative phosphorylation or TNF-α signaling among others. The ten genes with the strongest up- and downregulation of this gene set were validated by qPCR (Fig. 3E; Additional file 1: Figure S3A, Additional file 2: Table S5). In HCmel12 melanoma, the downregulation of a melanocyte differentiation gene set is associated with local tumor growth and lung metastasis [40]. But, among the cell lines used here a similar relation of this gene set with liver colonization could not be detected (Additional file 1: Figure S3B).

Overall, comparative gene expression analysis including heat maps of GOBP demonstrated that differences between the cell lines with different propensity for hepatic colonization are especially found in the processes of cell migration (Additional file 1: Figure S4) and angiogenesis (Fig. 3F, Additional file 1: Figure S5).

**Fig. 4:**
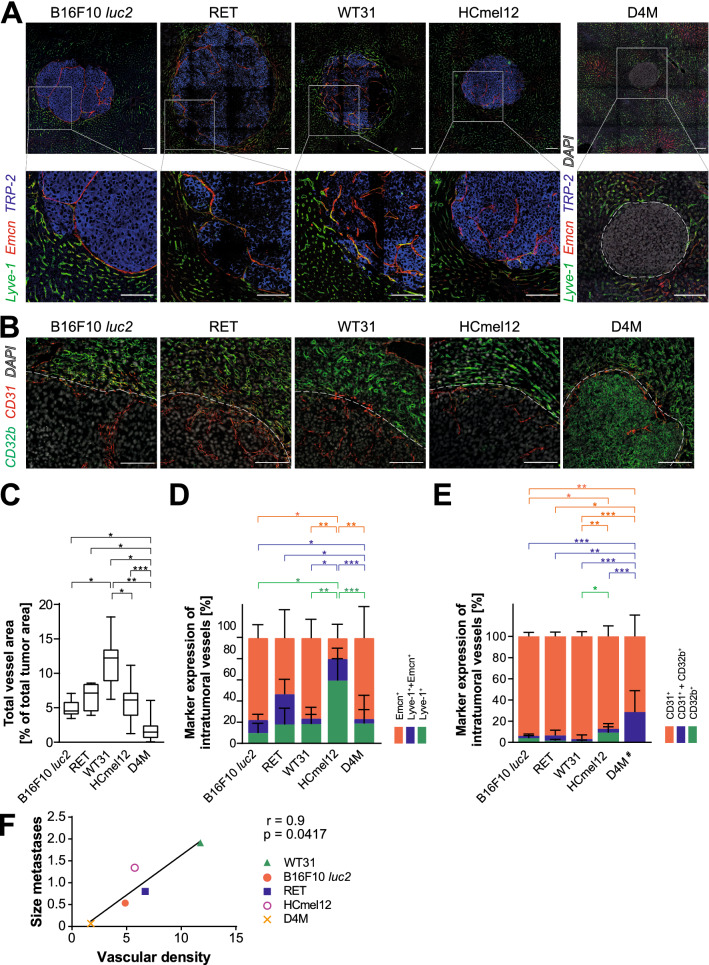
Melanoma metastases differ in vascular density. Intratumoral EC express continuous EC-associated marker proteins. **A** Images of tile scans of immunofluorescence stainings for Lyve-1, Emcn and TRP-2 of liver metastases of B16F10 *luc2*, RET, WT31, HCmel12 and D4M melanoma. Representative images are displayed. Scale bars = 100 µm, n ≥ 5. Detail magnifications of corresponding images show the border of hepatic metastasis to adjacent hepatic tissue. Scale bars = 100 µm. **B** Immunofluorescences for CD31, CD32b and DAPI of hepatic metastasis of B16F10 *luc2*, RET, WT31, HCmel12 and D4M melanoma at the border to adjacent liver. White dotted lines present the border of hepatic metastases to adjacent liver tissue. Scale bars = 100 µm, n ≥ 5. **C** Quantification of intratumoral blood vessels. Emcn^+^ area in relation to total tumor area is presented. A Dunn’s test was used (p = 0.0200 for B16F10 vs. WT31; p = 0.0105 for B16F10 vs. D4M; p = 0.0400 for RET vs. D4M; p = 0.0130 for WT31 vs. HCmel12; p < 0.0001 for WT31 vs. D4M; p = 0.0020 for HCmel12 vs. D4M). A box and whisker plot is presented with distribution of values from minimum to maximum. **D** Intratumoral EC were analyzed for Emcn or Lyve-1 marker expression. Corresponding marker expression was set in relation to total intratumoral vessel area (100%). Proportion of Lyve^+^, Lyve^+^  + Emcn^+^ as well as Emcn^+^ area is displayed. A Dunn’s test was applied. Red bars present the statistical comparisons of Emcn expression, blue bars of Lyve-1 and Emcn expression and green bars demonstrate statistical differences in Lyve-1 expression (Emcn: p = 0.0140 for B16F10 vs. HCmel12; p = 0.0020 for WT31 vs. HCmel12; p = 0.0010 for HCmel12 vs. D4M; Lyve-1 and Emcn: p = 0.0120 for B16F10 vs. D4M; p = 0.0440 for RET vs. D4M; p = 0.0170 for WT31 vs. HCmel12; p < 0.0001 for HCmel12 vs. D4M; Lyve-1: p = 0.0140 for B16F10 vs. HCmel12; p = 0.0080 for WT31 vs. HCmel12; p < 0.0001 for HCmel12 vs. D4M). Number of animals analyzed = 6 (B16F10 *luc2*), 3 (RET), 7 (WT31), 3 (HCmel12), 4 (D4M). Number of metastases analyzed = 6 (B16F10 *luc2*), 4 (RET), 10 (WT31), 10 (HCmel12), 25 (D4M). **E.** Expression of CD31 and CD32b of intratumoral vessels was analyzed (see E for IF images) and was set in relation to total intratumoral vessel area (100%). Proportion of CD31^+^, CD31^+^  + CD32b^+^ as well as CD32b^+^ area is displayed. A Dunn’s test was applied. Red bars present statistical comparison of CD31 expression, blue bars of CD31 and CD32b expression and whereas green bars demonstrate statistical differences in CD32b expression (CD31: p = 0.0140 for B16F10 vs. HCmel12; p = 0.0020 for WT31 vs. HCmel12; p = 0.0020 for B16F10 vs. D4M, p = 0.0210 for RET vs. D4M, p < 0.0001 for WT31 vs. D4M,; CD31 + CD32b: p < 0.0001 for B16F10 vs. D4M; p = 0.0080 for RET vs. D4M; p < 0.0001 for WT31 vs. D4M; p < 0.0001 for HCmel12 vs. D4M; CD32b: p = 0.0280 for WT31 vs. HCmel12). Number of animals analyzed = 4 (B16F10 *luc2*), 3 (RET), 5 (WT31), 4 (HCmel12), 3 (D4M). Number of metastases analyzed = 6 (B16F10 *luc2*), 4 (RET), 16 (WT31), 6 (HCmel12), 12 (D4M). **F** A Spearman’s correlation of the mean vascular density and the mean size of hepatic melanoma metastases was calculated among all melanoma cell lines (r = 0.9). A one-sided t-test was performed (p = 0.0417). Data information: *P < 0.05, **P < 0.01, *** P < 0.0001, n.s. = not significant.

To further investigate the angiogenic heterogeneity of the cell lines, the total vessel area of the metastases was analyzed (Fig. 4A). WT31 melanoma metastases were the most vascularized ones (11.8% ± 1.1), while hepatic metastases of D4M presented with the fewest vessels (1.7% ± 0.3), followed by B16F10 *luc2* (4.9% ± 0.5) (Fig. 4C). To assess the phenotype of intratumoral blood vessels, markers for continuous endothelial cells (ECs), such as Endomucin (Fig. 4A, D) and CD31 (Fig. 4B, E; Additional file 1: Figure S6A), or markers of Liver sinusoidal endothelial cells (LSEC), Lyve-1 (Fig. 4A, D), CD32b (Fig. 4B, E; Additional file 1: Figure S6A) or Stab2 (Additional file 1: Figure S7A), were analyzed. Strong expression of continuous EC markers and reduced expression of LSEC markers was seen in all hepatic metastases indicating a predominantly capillarized phenotype of intratumoral vessels (Fig. 4D, E). However, the percentages of Lyve1^+^ or CD32b^+^ intratumoral vessels were significantly higher in HCmel12 in comparison to the other melanoma metastases (Lyve1^+^: 59.9% ± 6.3; CD32b^+^: 9.2% ± 3.4) indicating a mixed sinusoidal and capillarized molecular phenotype in a minor fraction of HCmel12 vessels (Fig. 4D, E). There was a strong and significant correlation of increasing metastasis size with vascular density of the different melanomas (Fig. 4F). Altogether vascularization tended to correlate with increasing efficiency and intratumoral vessels showed a predominant continuous endothelial cell phenotype.

**Fig. 5:**
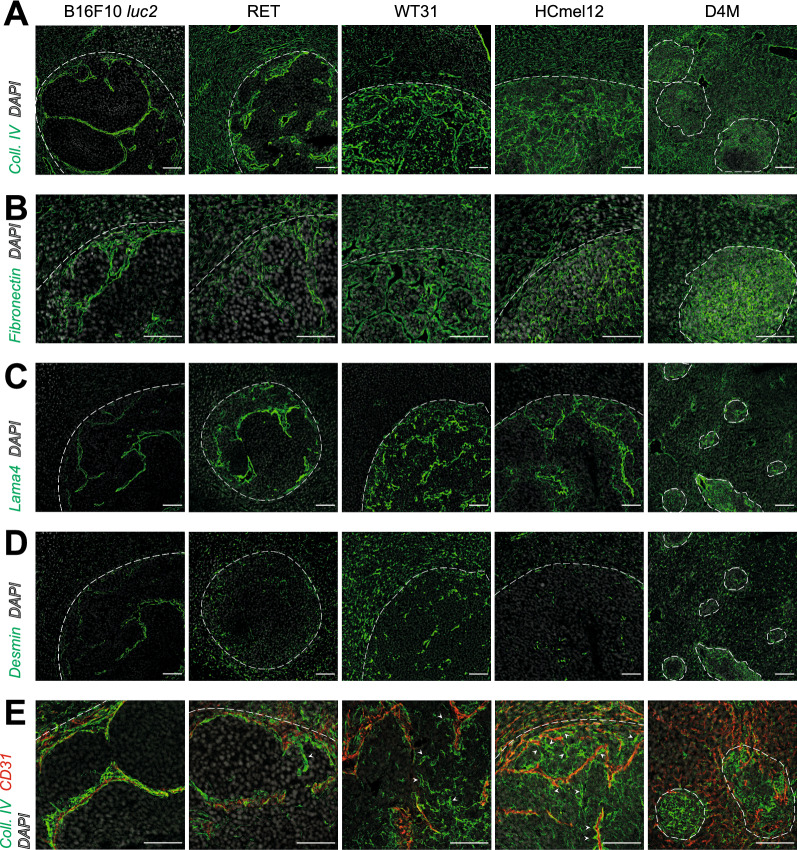
Analysis of extracellular matrix protein deposition in hepatic melanoma metastases. **A** Immunofluorescences of Collagen IV and DAPI of livers with hepatic melanoma metastases of B16F10 *luc2*, RET, WT31, HCmel12 and D4M. White dotted lines present the border of hepatic metastases to adjacent liver tissue. Scale bars = 100 µm, n ≥ 5. **B** Images of immunofluorescence stainings for Fibronectin and DAPI of liver metastases of B16F10 *luc2*, RET, WT31, HCmel12 and D4M melanoma. White dotted lines present the border of hepatic metastases to adjacent liver tissue. Scale bars = 100 µm, n ≥ 5. **C** Immunofluorescences for Lama4 of B16F10 *luc2*, RET, WT31, HCmel12 and D4M melanoma liver metastases. White dotted lines present the border of hepatic metastases to adjacent liver tissue. Scale bars = 100 µm, n ≥ 5. **D** Immunofluorescences for Desmin of hepatic B16F10 *luc2*, RET, WT31, HCmel12 and D4M melanoma metastases. White dotted lines present the border of hepatic metastases to adjacent liver tissue. Scale bars = 100 µm, n ≥ 5. **E** Pictures of immunofluorescence stainings for Collagen IV, CD31 and DAPI of hepatic melanoma metastases of B16F10 *luc2*, RET, WT31, HCmel12 and D4M. White dotted lines present the border of hepatic metastases to adjacent liver tissue. Besides, white arrows highlight structures indicative of empty sleeves. Scale bars = 100 µm, n ≥ 5

To further characterize vessel maturation and differentiation in the formed metastases, the deposition of extracellular matrix proteins surrounding the intratumoral endothelium was assessed. Strong subendothelial deposition of Collagen IV (Fig. 5A), Collagen I (Additional file 1: Figure S7B), Collagen III (Additional file 1: Figure S7C), Fibronectin (Fig. 5B) and Laminin-α4-integrin (Lama4) (Fig. 5C) was observed in all metastases indicating a continuous phenotype with basement membrane formation. Likewise, Desmin^+^ periendothelial cells were similarly present in all metastases indicating similar coverage by pericytes (Fig. 5D). Remarkably, Collagen IV expression outside the perivascular space was seen in metastases of WT31, HCmel12 and D4M (Fig. 5A). In highly vascularized WT31 and HCmel12 these Collagen IV ^+^ structures appeared sleeve-like. This indicates high angiogenic activity with so called empty sleeves as they lack direct association with CD31^+^ EC [41]. Collagen IV^+^ CD31^−^ empty vessel sleeves were far more frequent in WT31 and HCmel12 in comparison to the other cell lines suggesting highly active but partially inefficient angiogenesis (Fig. 5E; Additional file 1: Figure S6B). Overall, high vascularization and angiogenic activity appeared to relate with larger hepatic metastatic size and lower propensity to tumor necrosis.

**Fig. 6:**
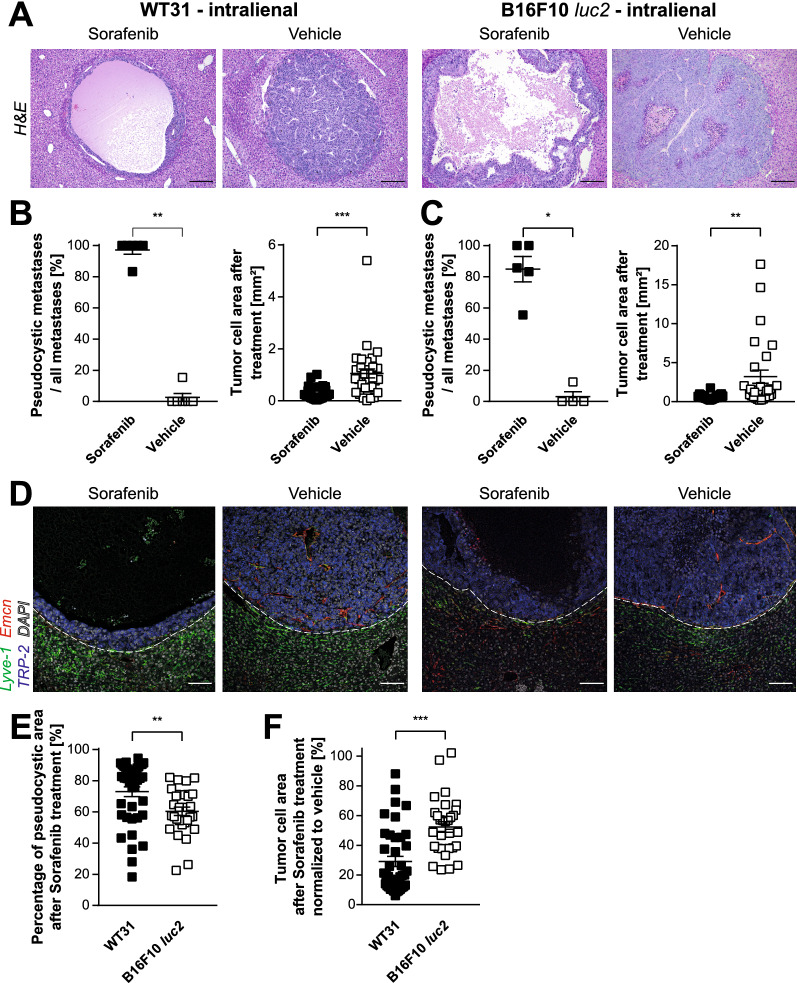
Treatment with Sorafenib leads to pseudocystic hemorrhagic degeneration and loss of intratumoral vessels of hepatic melanoma metastases. WT31 melanoma cells (0.3 × 10^5^ cells) or B16F10 *luc2* melanoma cells (1.5 × 10^5^ cells) were injected intrasplenically. From day 1 to 18 mice received daily i.p. injections of Sorafenib (60 mg/kg KG) or vehicle controls (solvent controls). At day 19 mice were sacrificed. Number of animals/ group = 6 (WT31, Sorafenib), 7 (WT31, Vehicle), 6 (B16F10 *luc2*, Sorafenib), 6 (B16F10 *luc2*, Vehicle). **A** Pictures of H&E stainigs of metastases of WT31 (left panels) or B16F10 *luc2* melanoma (right panels) of mice treated with Sorafenib or vehicle. Scale bar = 200 µm, n ≥ 5. **B** The percentage of pseudocystic metastases in relation to the total number of metastases was quantified for WT31 melanoma treated with Sorafenib or vehicle. Analysis is shown per animal. A Mann–Whitney U-test was performed (p = 0.0022). Number of animals analyzed = 6 (WT31, Sorafenib), 6 (WT31, Vehicle). Number of metastases analyzed = 48 (WT31, Sorafenib), 32 (WT31, Vehicle). Besides, the tumor cell area of WT31 metastases in mice treated with Sorafenib or vehicle was measured. A Mann–Whitney U-test was performed (p < 0.0001). Number of animals analyzed = 6 (WT31, Sorafenib), 6 (WT31, Vehicle). Number of metastases analyzed = 41 (WT31, Sorafenib), 32 (WT31, Vehicle). **C** The percentage of pseudocystic metastases in relation to the total number of metastases was quantified for B16F10 *luc2* melanoma treated with Sorafenib or vehicle. Analysis is shown per animal. A Mann–Whitney U-test was performed (p = 0.0159). Number of animals analyzed = 5 (B16F10 *luc2*, Sorafenib), 4 (B16F10 *luc2*, Vehicle). Number of metastases analyzed = 39 (B16F10 *luc2*, Sorafenib), 28 (B16F10 *luc2*, Vehicle). Moreover, the tumor cell area of B16F10 *luc2* metastases in mice treated with Sorafenib or vehicle was measured. A Mann–Whitney U-test was performed (p = 0.0002). Number of animals analyzed = 5 (B16F10 *luc2*, Sorafenib), 4 (B16F10 *luc2*, Vehicle). Number of metastases analyzed = 39 (B16F10 *luc2*, Sorafenib), 28 (B16F10 *luc2*, Vehicle). **D** Immunofluorescences of Lyve-1, Emcn, TRP-2 and DAPI of livers with hepatic melanoma metastases of WT31 (left panels) or B16F10 *luc2* melanoma (right panels) treated either with Sorafenib or vehicle. White dotted lines present the border of hepatic metastases to adjacent liver tissue. Scale bars = 100 µm, n ≥ 5. **E** The pseudocystic area was set in relation to the total size of WT31 or B16F10 *luc2* metastases that were treated with Sorafenib. A Mann–Whitney U-test was performed (p = 0.0004). Number of animals analyzed = 6 (WT31, Sorafenib), 5 (B16F10 *luc2*, Sorafenib). Number of metastases analyzed = 40 (WT31, Sorafenib), 30 (B16F10 *luc2*, Vehicle). **F** The response to Sorafenib was determined as percentage of tumor cell area in the Sorafenib group and normalized to the percentage of tumor cell area of vehicle controls. Normalization needs to be performed because of variable size of metastases, necrotic or cystic areas in the vehicle control group. A Mann–Whitney U-test was performed (p < 0.0001). Number of animals analyzed = 6 (WT31, Sorafenib), 5 (B16F10 *luc2*, Sorafenib). Number of metastases analyzed = 40 (WT31, Sorafenib), 30 (B16F10 *luc2*, Vehicle). Data information: *P < 0.05, **P < 0.01, *** P < 0.0001, n.s. = not significant

To assess whether these different patterns of vascularization also translate into differing treatment responses to anti-angiogenic treatment, highly vascularized WT31 and poorly vascularized B16F10 *luc2* were treated with Sorafenib, an anti-angiogenic multi-kinase inhibitor, or a corresponding solvent control (vehicle). In both models Sorafenib led to central pseudocystic, hemorrhagic degradation of metastases with a thin residual rim of viable tumor cells in around 80–90% of metastases (Fig. 6A–C, Additional file 1: Figure S8A). Likewise, WT31 and B16F10 *luc2* showed a significantly decreased tumor cell area and almost complete abolishment of intratumoral blood vessels after Sorafenib treatment (Fig. 6B–D). However, comparison between WT31 and B16F10 *luc2* indicated that treatment with Sorafenib is indeed less effective in poorly vascularized B16F10 *luc2.* The reduction of the viable tumor cell area was significantly larger in WT31 (70.9% vs. 52.3% reduction) and similar the induction of pseudocystic degradation was significantly higher in WT31 (73% vs. 60.3%) indicating stronger dependence on vascularization than B16F10 *luc2* (Fig. 6E, F). In addition, there was a trend to significance (p = 0.1970) that the fraction of metastases showing degradation was higher in WT31 (97.2% ± 2.8) than B16F10 *luc2* (84.92% ± 8.1) (Additional file 1: Figure S8C). In summary, these data show that hepatic metastases of highly and poorly vascularized murine melanomas significantly respond to anti-angiogenic treatment with Sorafenib. Of note, this response is even stronger in highly vascularized lesions in comparison to poorly vascularized ones.

## Discussion

Hepatic metastasis was recently shown to be a decisive negative factor for the response to immunotherapy in patients with melanoma[7]. Therefore, there is an urgent need for preclinical models to study the underlying mechanisms driving organ-specific melanoma metastasis to the liver and to develop novel therapeutic approaches. Genetic mouse models to study spontaneous melanoma metastasis are scarce [42] and hepatic metastasis usually only occurs at a very low frequency [27, 29, 43, 44]. Thus, genetic models are currently not feasible in this context. Spleen injection of various tumor cells including melanoma cells is an established method to investigate liver colonization [31, 45]. Here we show that this model can reliably be used to comparatively study efficiency, morphologic and molecular features as well as tumor angiogenesis and treatment responses of hepatic metastases of different melanoma cells.

WT31 melanoma cells stood out with the highest efficiency of hepatic colonization after intrasplenic injection. In addition, they consistently established metastases to the liver and other organs in a CM-like pattern after intravenous injection. These two techniques provide ideal and reliable settings to study hepatic melanoma colonization either with or without simultaneous metastasis to other organs. WT31 is the only cell line investigated by us with a human NRAS mutation [28]. Although human BRAF- and NRAS-mutated melanomas have been associated with an increased risk for liver metastases, this association was rather weak [24] and until now it has not been shown that one of these main driver mutations is a key determinant of hepatic metastasis. This notion is also supported by the fact that D4M, which harbor a BRAF mutation, showed the lowest efficiency and smallest metastatic sizes in our analysis. Nonetheless, the mutational landscape of melanoma clearly influences organ-specific metastasis as KRAS driver mutations were recently associated with brain colonization as first site of metastasis [46]. Although, the mutational landscape of human melanoma with liver metastases has not been specifically characterized, it can be assumed that hepatotropism of melanoma is controlled in multifactorial fashion and involves non-genetic and epigenetic traits.

To more comprehensively study the molecular differences that may underly the heterogeneity of hepatic melanoma metastasis in our study, all melanoma cell lines were analyzed by RNAseq. Differential transcriptomic analysis of high and intermediate metastatic (HIM-) melanoma compared to low metastatic (LM-) melanoma showed the most marked differences in pathways involved in cell migration, angiogenesis, EMT, oxidative phosphorylation or TNF-α signaling. Cell adhesion and migration are the first steps of organ colonization in the metastatic cascade. In this regard, integrin alpha2 is associated with enhanced hepatic colonization while integrin alpha4 is associated with lymph node metastasis in B16 melanoma [25, 47, 48]. Although integrin alpha2 was inconsistently expressed among cell lines of HIM- and LM-melanoma, WT31 melanoma was the only cell line with strong integrin alpha4 expression which may contribute to its high metastatic efficiency. MSX1 which has been shown to increase hepatic melanoma metastasis in an immune-deficient setting [26] was only expressed in D4M melanoma and therefore could not account for the differences observed by us (data not shown). Overall, cell migration is likely a major determinant of organ-specific metastatic efficiency and appears to be controlled by complex gene sets. This notion is also supported by the fact that expression of gene sets involved in cell adhesion can be used as predictors of lymph node metastasis [49]. Therefore, there is an urgent need for studies identifying crucial gene sets in melanoma patients with liver metastasis to select patients at risk early in the course.

Angiogenesis also stood out as a differentially regulated process between HIM- and LM-melanoma. Angiogenesis is well known as determinant of hepatic metastasis in general [50]. In CRC the stromal subtype with an angiogenic signature shows enhanced liver metastasis [17]. The critical role of angiogenesis for hepatic metastasis of CRC is further supported by a molecular analysis of liver metastases and their corresponding primary tumors [51]. Liver metastases of breast cancer, CRC, UM and CM share three common main HGPs with different tumor vessels: desmoplastic, replacement and pushing type [11–14]. Desmoplastic and pushing type HGP strongly rely on de-novo angiogenesis of tumor vessels whereas replacement HGP co-opts the pre-existing sinusoidal liver vasculature. Paku et al. postulated cooperation of smooth muscle actin-positive cells and fusion of partly capillarized sinusoids as hallmark of angiogenesis in pushing type HGPs [52]. In our model pushing type HGPs were found in all metastases and supportive connective tissue was detected in all types supporting the hypothesis of Paku. Transdifferentiation of liver sinusoidal endothelial cells to a capillarized phenotype is also observed in tumor endothelium of murine and human hepatocellular carcinoma [53]. Since the melanoma cell lines with higher vascularization developed larger hepatic metastases, efficiency of hepatic melanoma colonization and outgrowth of metastases are strongly regulated by angiogenic processes.

Aside from angiogenesis itself, vessel maturity which is regulated by vessel pruning and regression is an important process involved in tumor blood supply [41]. Detection of empty vessel sleeves as sign for vessel pruning and regression showed that both WT31 and HCmel12 melanoma metastases have high angiogenic activities. Although HCmel12 exhibited a partly sinusoidal phenotype of tumor EC, the vessel morphology did not imply co-option as seen in replacement HGP.

The tumor vasculature can be disrupted by anti-angiogenic agents such as bevacizumab, an anti-VEGF antibody, or Sorafenib, a multi-kinase-inhibitor [14, 54]. First approaches with such therapeutic agents were disappointing and clinical studies in patients with advanced melanoma were usually terminated in early phases [55]. However, clinical studies with Sorafenib did not investigate treatment responses in subgroups of patients with hepatic metastasis. In addition, in these patients it may be necessary to stratify for HGPs or tumor vascularization patterns and to treat in combination or sequentially with current standard of care treatments. In our study, Sorafenib induced reliable pseudocystic degeneration in high metastatic melanoma (WT31) and in intermediate metastatic melanoma (B16F10 *luc2*). Although this response was even stronger in WT31, both cell lines showed a solid treatment response. Necrosis or pseudocystic degeneration is not seen when subcutaneous B16 melanoma is treated with Sorafenib [56]. This indicates that the outcome of Sorafenib treatment and anti-angiogenesis depends on the organ-specific microenvironment. In this context lower oxygen levels in the liver may be a contributing factor. Like anti-angiogenic treatment in desmoplastic CRC [57] a small rim of viable tumor cells was still present after treatment of our models. This indicates that a combinatorial approach is likely necessary to achieve complete remission of hepatic metastasis. Due to the subtle effect of chemotherapeutics alone and the missing improvement of therapy response to Sorafenib when combined with Dacarbazine [58], a combination of standard of care ICI or targeted therapy with anti-angiogenic drugs seems appealing for hepatic metastasis. This approach is particularly promising as a breakthrough in the therapy of hepatocellular carcinoma was achieved by the combination of atezolizumab and bevacizumab replacing the former gold standard Sorafenib [59].

Altogether, molecular and phenotypic diversity of murine melanoma determine liver metastatic propensity involving cell migration and angiogenesis as major processes. Metastatic vascularization correlates with metastatic size. Interestingly, both highly and poorly vascularized hepatic lesions responded to Sorafenib indicating a broad efficacy for Sorafenib in this specific context. Therefore, anti-angiogenic therapy is an appealing approach to treat liver metastasis in advanced melanoma patients.

## Conclusions

Heterogeneity of hepatic metastasis of cutaneous melanoma was studied using a murine orthotopic model with five genetically different cell lines. Efficacies and phenotypic features of liver colonization of these melanoma cell lines were comprehensively compared. Migration and angiogenesis were among the differentially regulated functions identified by comparative RNA-seq analysis. Overall, molecular and phenotypic heterogeneity of hepatic melanoma metastasis revealed angiogenesis as a targetable determinant of hepatic colonization paving the way for organ-specific anti angiogenic treatment.

## Supplementary Information

**Additional file 1:** **Figure S1.** Morphological characterization of melanoma cell lines. **A. **A selection of representative pictures of hepatic metastases of WT31, B16F10 *luc2*, RET, D4M and HCmel12 melanoma after spleen injection. Besides, liver metastases of WT31 melanoma after i.v. injection are shown. Scale bar = 1 cm. **B. **Images of cell pellets of B16F10 *luc2*, RET, WT31, HCmel12 and D4M melanoma. **C.** Representative pictures of lungs, brain, tibia, kidneys and spleen with metastases after intravenous injection of WT31 melanoma. Scale bar = 1 cm. **Figure S2.** Sirius red and PAS stainings of hepatic metastases of B16F10 *luc2*, RET, WT31, HCmel12 and D4M. **A. **Images of Sirius red (SR) stainings of hepatic metastases of B16F10 *luc2*, RET, WT31, HCmel12 and D4M melanoma. Scale bar = 200 µm, n ≥ 5. **B.** Pictures of liver metastases of B16F10 *luc2*, RET, WT31, HCmel12 and D4M melanoma stained by PAS. Scale bar = 200 µm, n ≥ 5. **Figure S3.** Heat map of top25 up- and downregulated genes and heat map of melanocytic differentiation genes. **A. **A heatmap of the 25 most up- and downregulated genes of HIM-melanoma in comparison to LM-melanoma is shown. Please also refer to Fig. 3A, B for definition of this gene set. **B.** A heat map of common melanocytic differentiation genes is presented. A red color code presents an upregulation of genes, whereas the green color code shows downregulation. Clustering of genes is presented on the left side. **Figure S4.** Heat map GOBP—cell migration. A heat map of the GOBP cell migration gene set is presented for B16F10, RET, WT31, HCmel12 and D4M melanoma cells. The heat map was split in two parts to fit the page. A red color code presents an upregulation of genes, whereas the green color code shows downregulation. Clustering of genes is presented on the left side. **Figure S5.** Heat map GOBP—angiogenesis. A heat map of the GOBP angiogenesis gene set is presented for B16F10, RET, WT31, HCmel12 and D4M melanoma cells. A red color code presents an upregulation of genes, whereas the green color code shows downregulation. Clustering of genes is presented on the left side. **Figure S6. A.** Additional pictures of immunofluorescences for CD31, CD32b and DAPI of hepatic metastasis of B16F10 *luc2*, RET, WT31, HCmel12 and D4M melanoma at the border to adjacent liver. Scale bars = 100 µm, n ≥ 5. **B.** Additional pictures of immunofluorescences for Collagen IV, CD31 and DAPI of hepatic metastasis of B16F10 *luc2*, RET, WT31, HCmel12 and D4M melanoma at the border to adjacent liver. Scale bars = 100 µm, n ≥ 5. **Figure S7.** Analysis of peri- and intratumoral Stab2 expression as well as extracellular Collagen I and Collagen III matrix deposition in hepatic metastases of B16F10 *luc2*, RET, WT31, HCmel12 and D4M melanoma. **A.** Immunofluorescence images of Stab2 and DAPI of B16F10 *luc2*, RET, WT31, D4M and HCmel12 melanoma liver metastases at the metastatic border. White dotted lines present the border of hepatic metastases to adjacent liver tissue. Scale bars = 100 µm, n ≥ 5. **B.** Immunofluorescences of Collagen I of hepatic metastases of B16F10 *luc2*, RET, WT31, HCmel12 and D4M melanoma. Scale bar = 100 µm, n ≥ 5. **C.** Immunofluorescences of Collagen III of liver metastases of B16F10 *luc2*, RET, WT31, HCmel12 and D4M melanoma. Scale bar = 100 µm, n ≥ 5. **Figure S8.** Treatment with Sorafenib leads to loss intratumoral vessels and cystic degeneration of hepatic melanoma metastases. **A.** WT31 melanoma cells (0.3 × 10^5^ cells) or B16F10 *luc2* melanoma cells (1.5 × 10^5^ cells) were injected intrasplenically. From day 1 to 18 mice received daily i.p. injections of Sorafenib (60 mg/kg KG) or vehicle/solvent controls. At day 19 mice were sacrificed. Macroscopic visible metastases were quantified. A Mann-Whitney Utest was performed (WT31: p = 0.0043; B16F10 *luc2*: p = 0.0745). Representative images are shown. Scale bars = 1 cm. Number of animals analyzed = 8 (WT31, Sorafenib), 9 (WT31, Vehicle), 6 (B16F10 *luc2*, Sorafenib), 6 (B16F10, Vehicle). **B.** The size of hepatic metastases of WT31 (left graph) or B16F10 *luc2* melanoma (right panel) in mice treated with Sorafenib or vehicle was measured. Mann-Whitney U-tests were performed (WT31: p = 0.5207; B16F10: p = 0.0326). Number of animals analyzed = 6 (WT31, Sorafenib), 6 (WT31, Vehicle), 5 (B16F10 *luc2*, Sorafenib), 4 (B16F10, Vehicle). Number of metastases analyzed = 48 (WT31, Sorafenib), 32 (WT31, Vehicle), 57 (B16F10 *luc2*, Sorafenib), 28 (B16F10, Vehicle). **C.** Comparison of the percentage of pseudocystic WT31 or B16F10 *luc2* melanoma metastases after treatment with Sorafenib. MannWhitney U-tests were performed (p = 0.1970). Number of animals analyzed = 6 (WT31, Sorafenib), 5 (B16F10 *luc2*, Sorafenib). Data information: *P < 0.05, **P < 0.01, *** P < 0.0001, n.s. = not significant.

**Additional file 2: Table S1.** Statistic analysis of metastatic efficiency and number of hepatic metastases of B16F10 *luc2*, RET, WT31, HCmel12 and D4M after spleen injection. **Table S2. A.** Percentage of animals with hepatic metastasis after spleen injections of melanoma cells. **B.** Number of hepatic metastases of each individual animal after spleen injections of melanoma cells. **Table S3. A.** Percentage of animals with hepatic metastasis after intravenous injections of WT31 melanoma cells. **B.** Number of hepatic metastases of each individual animal after intravenous injections of WT31 melanoma cells. **Table S4.** Sequences of primers used for qPCRs. **Table S5.** Statistic analysis of qPCRs of Kazn, St6galnac3, Glrb, Tyr, Unc5c, Thbs2, Tfap2b, Tenm3, Stambpl1 and Ly6e in WT31, B16F10 *luc2*, RET, D4M and HCmel12 melanoma cells.

## Data Availability

The raw and normalized gene expression profiling data have been deposited in NCBI's Gene Expression Omnibus and are accessible through GEO Series accession number GSE185539.

## Abbreviations

BRAFi: BRAF inhibitor
CM: Cutaneous melanoma
CRC: Colorectal carcinoma
HGP: Histological growth pattern
HIM-melanoma: High and intermediate metastatic melanoma
ICI: Immune checkpoint inhibition
Lama4: Laminin- α4-integrin
LM-melanoma: Low metastatic melanoma
LSEC: Liver sinusoidal endothelial cells
MEKi: MEK inhibitor
UM: Uveal melanoma
