# Supplementary material for: Angiogenic and molecular diversity determine hepatic melanoma metastasis and response to anti-angiogenic treatment
Source: J Transl Med. 2022 Feb 2;20:62. doi: 10.1186/s12967-022-03255-4 (PMC8812268; doi:10.1186/s12967-022-03255-4)

Suppl. Figure 1

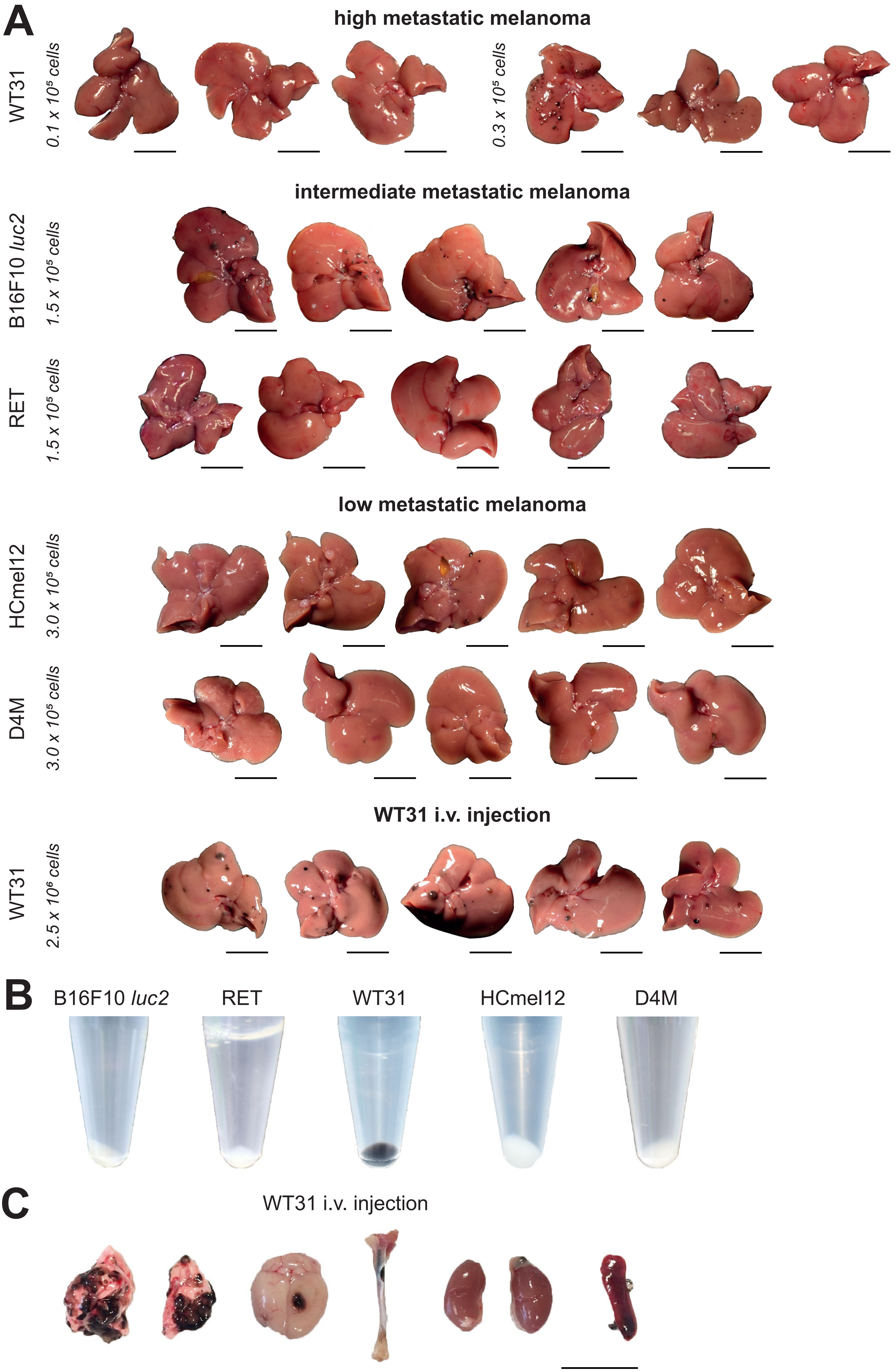

Suppl. Figure 2

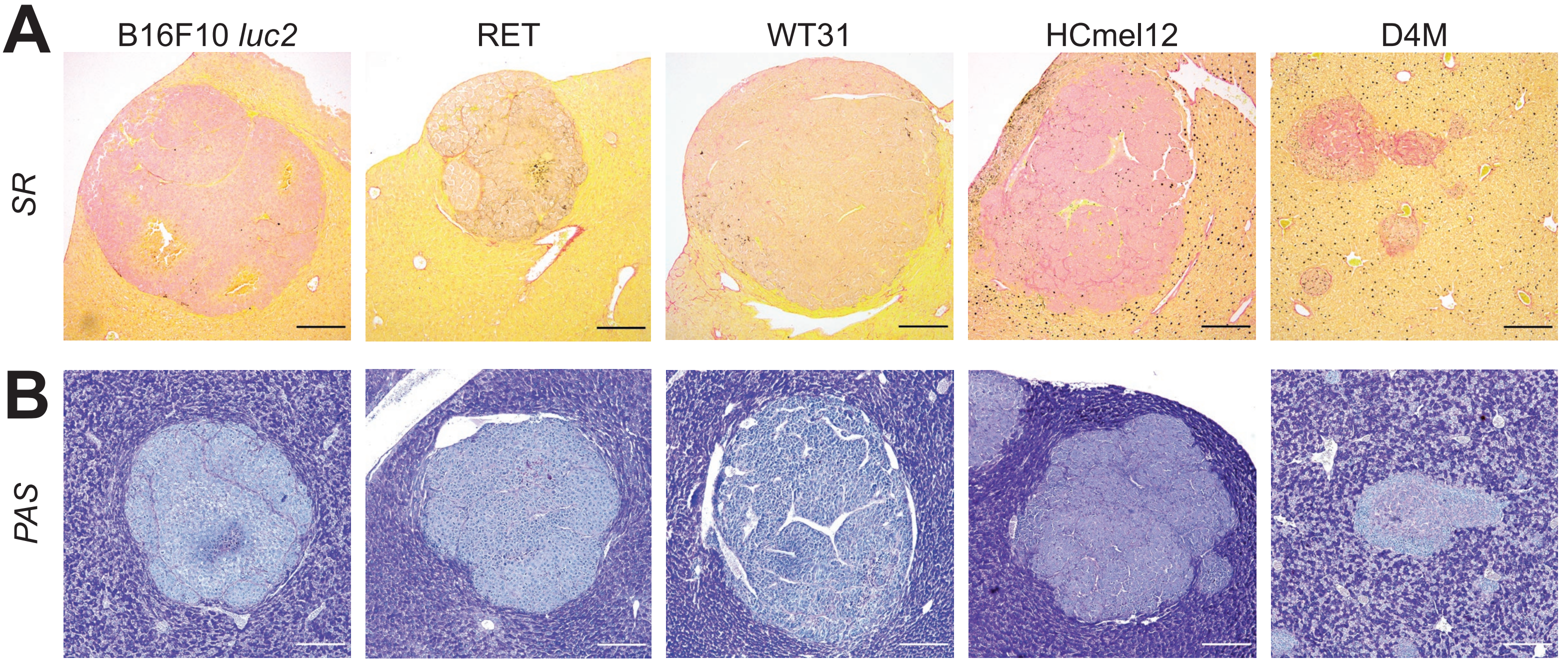

Suppl. Figure 3

A

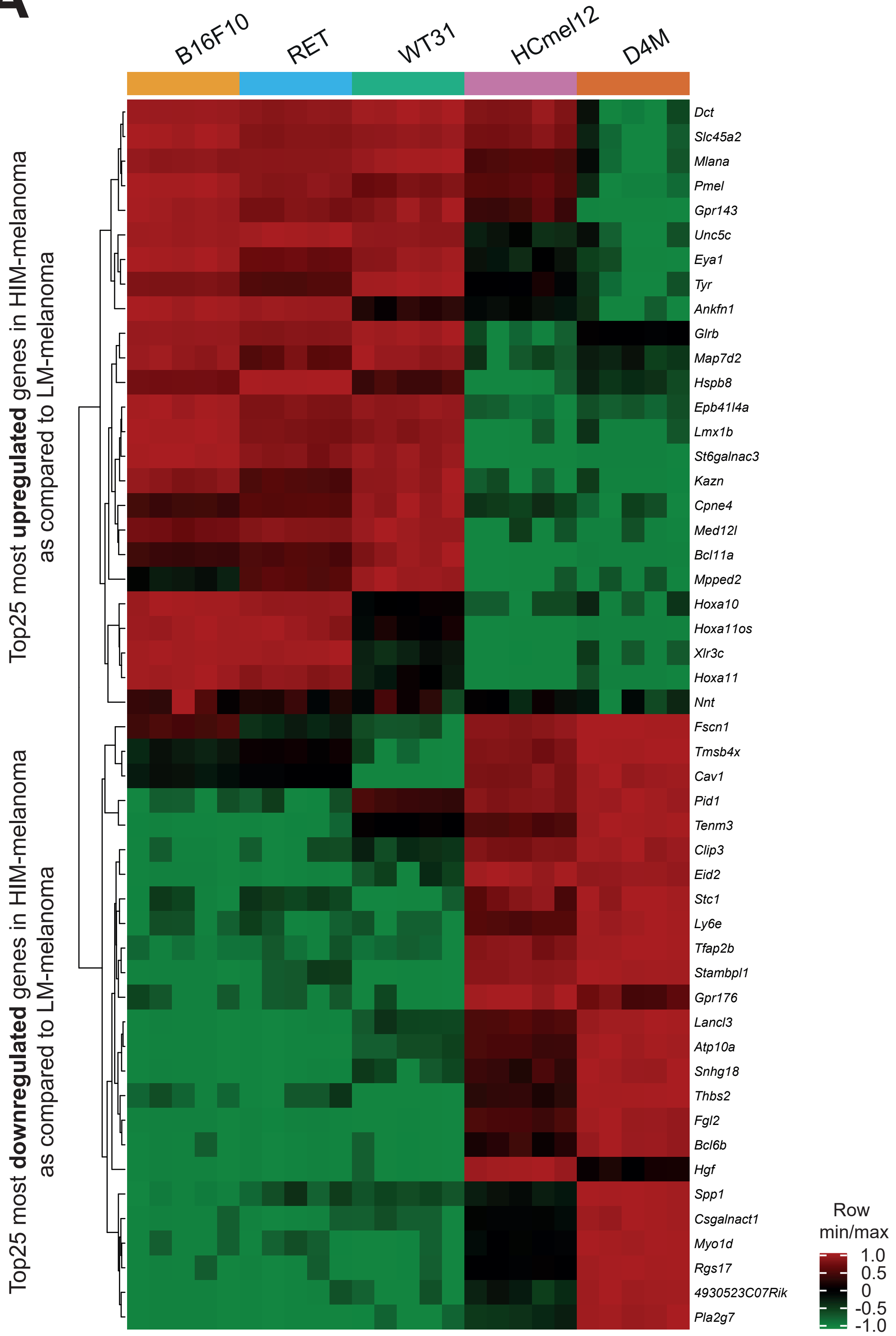

B

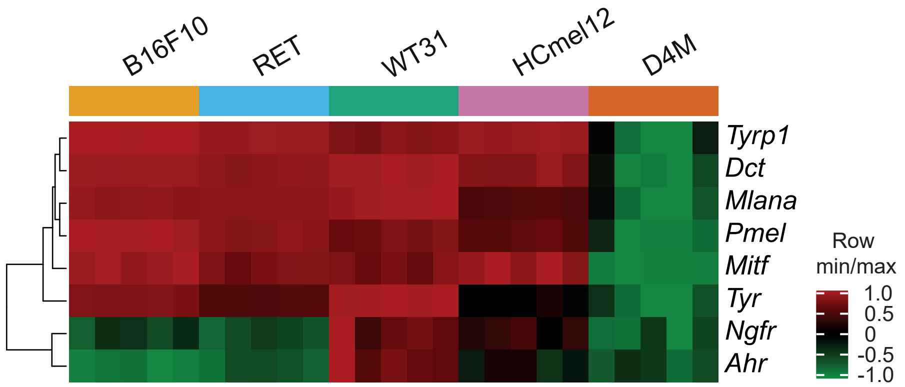

Suppl. Figure 4

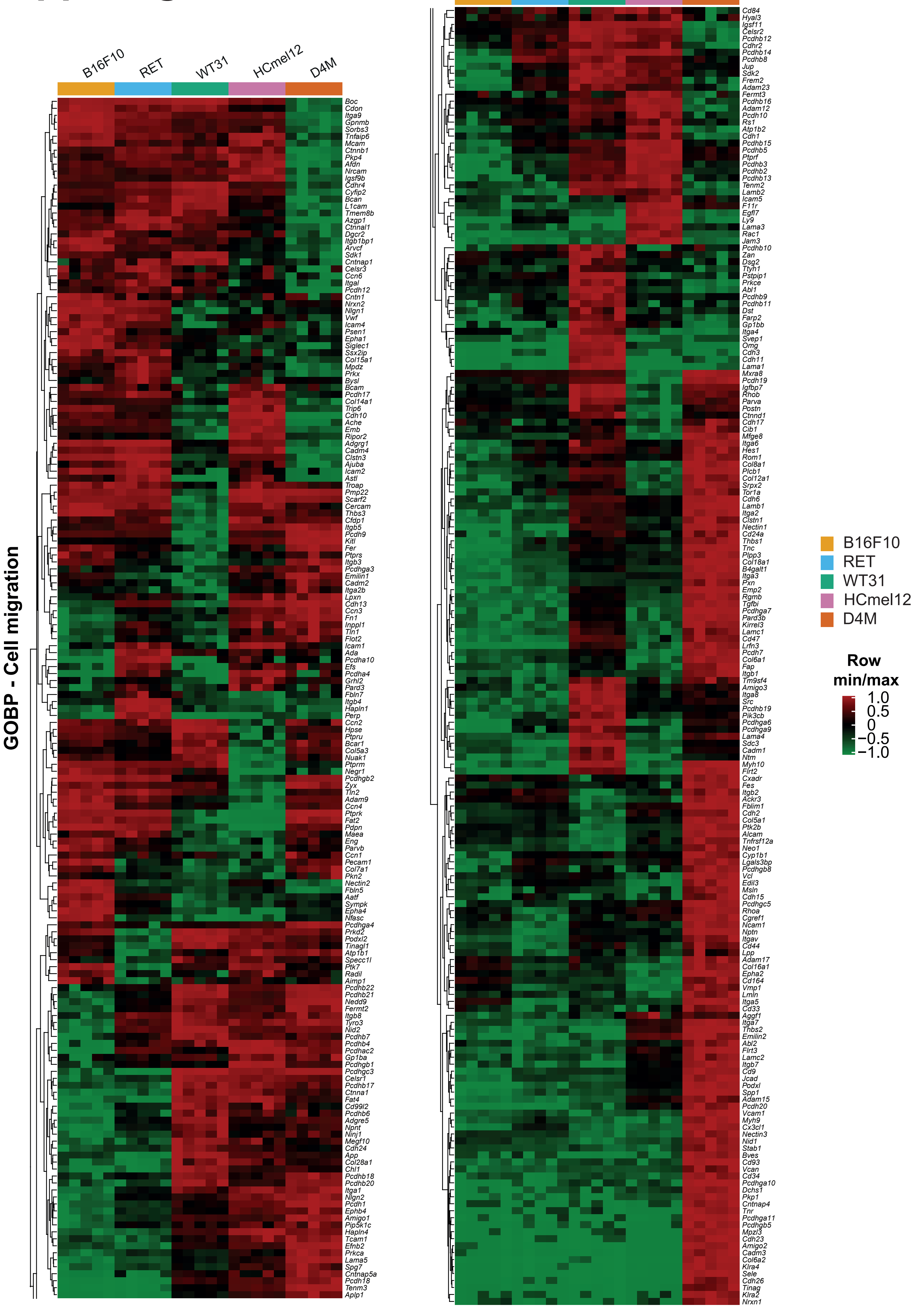

Suppl. Figure 5

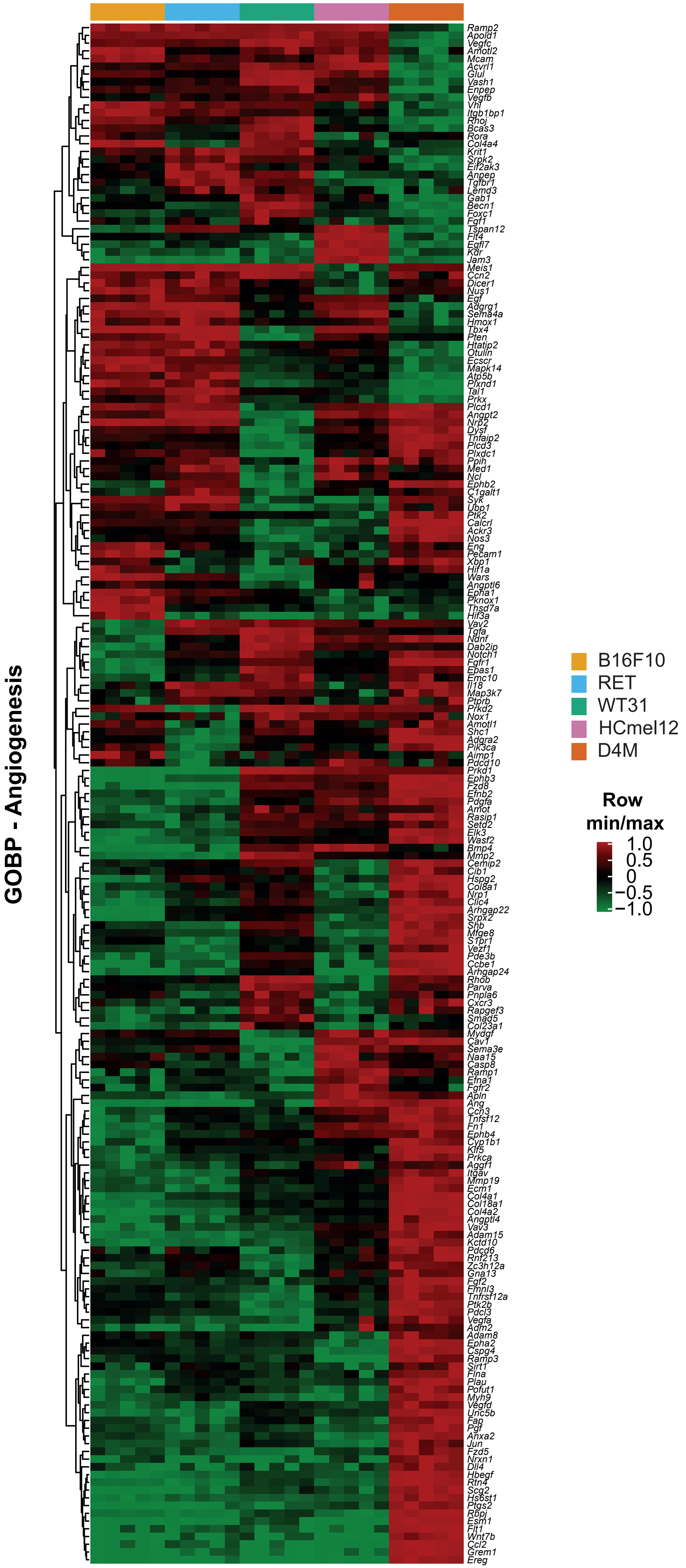

Suppl. Figure 6

A

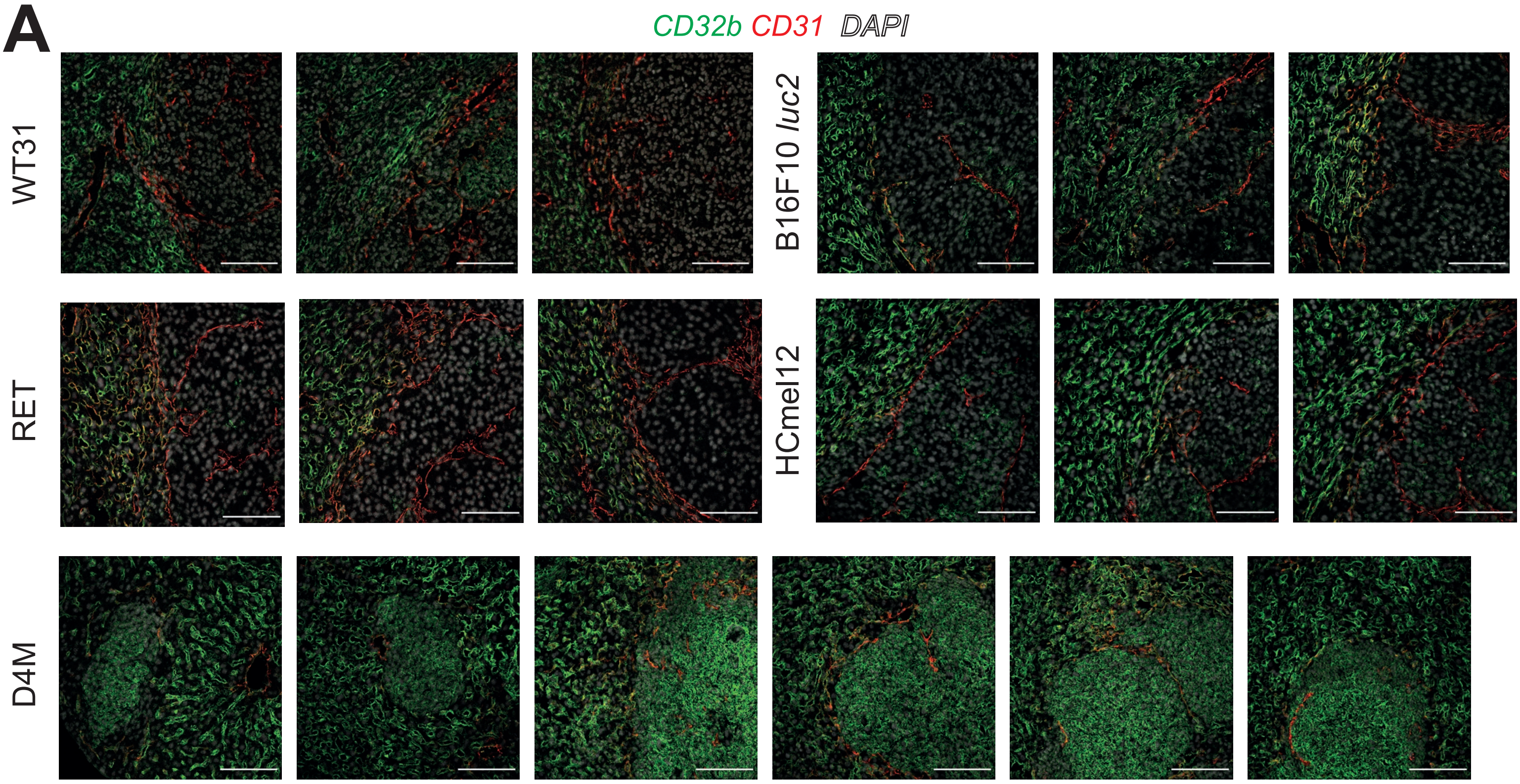

B

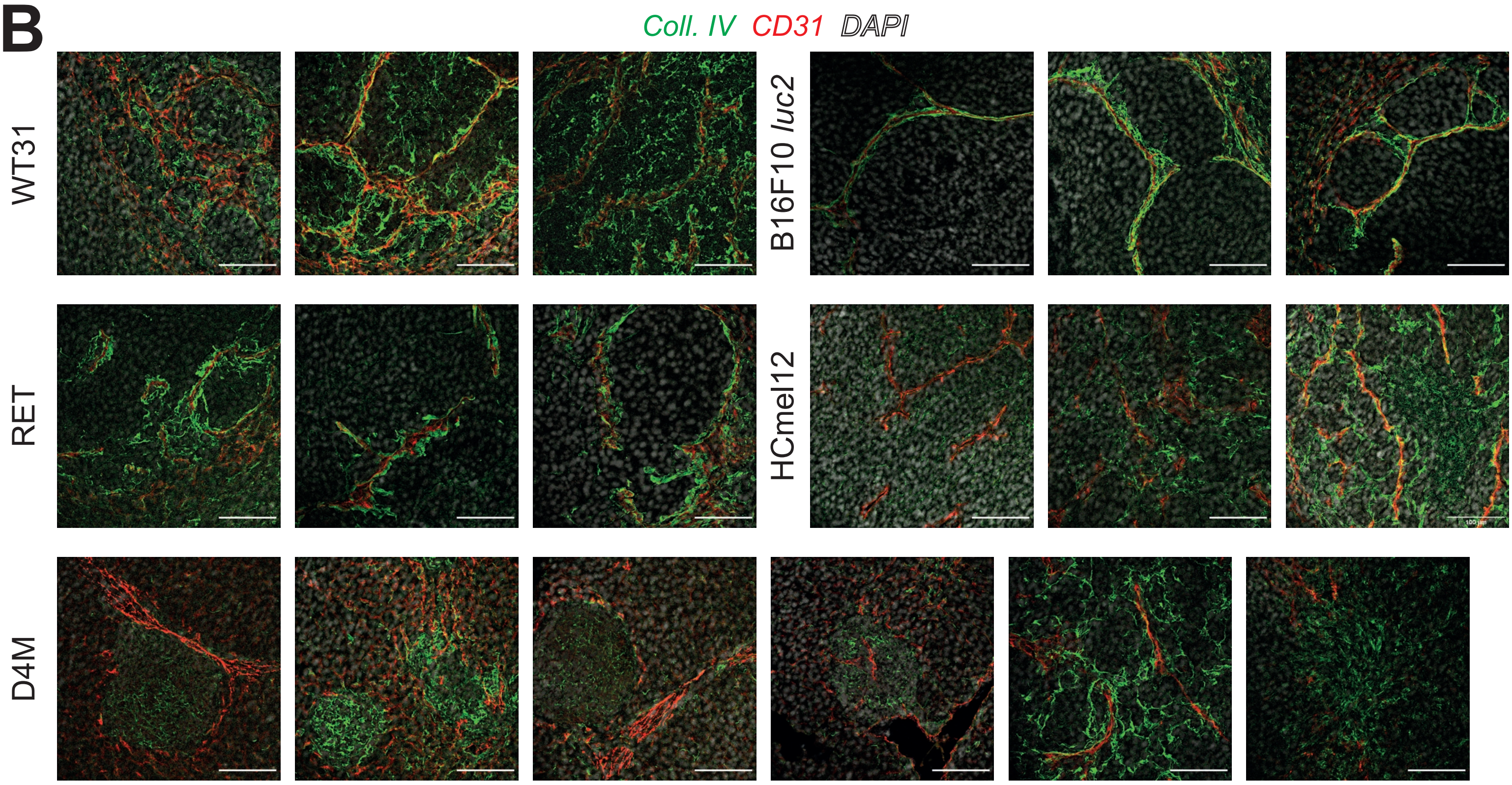

Suppl. Figure 7

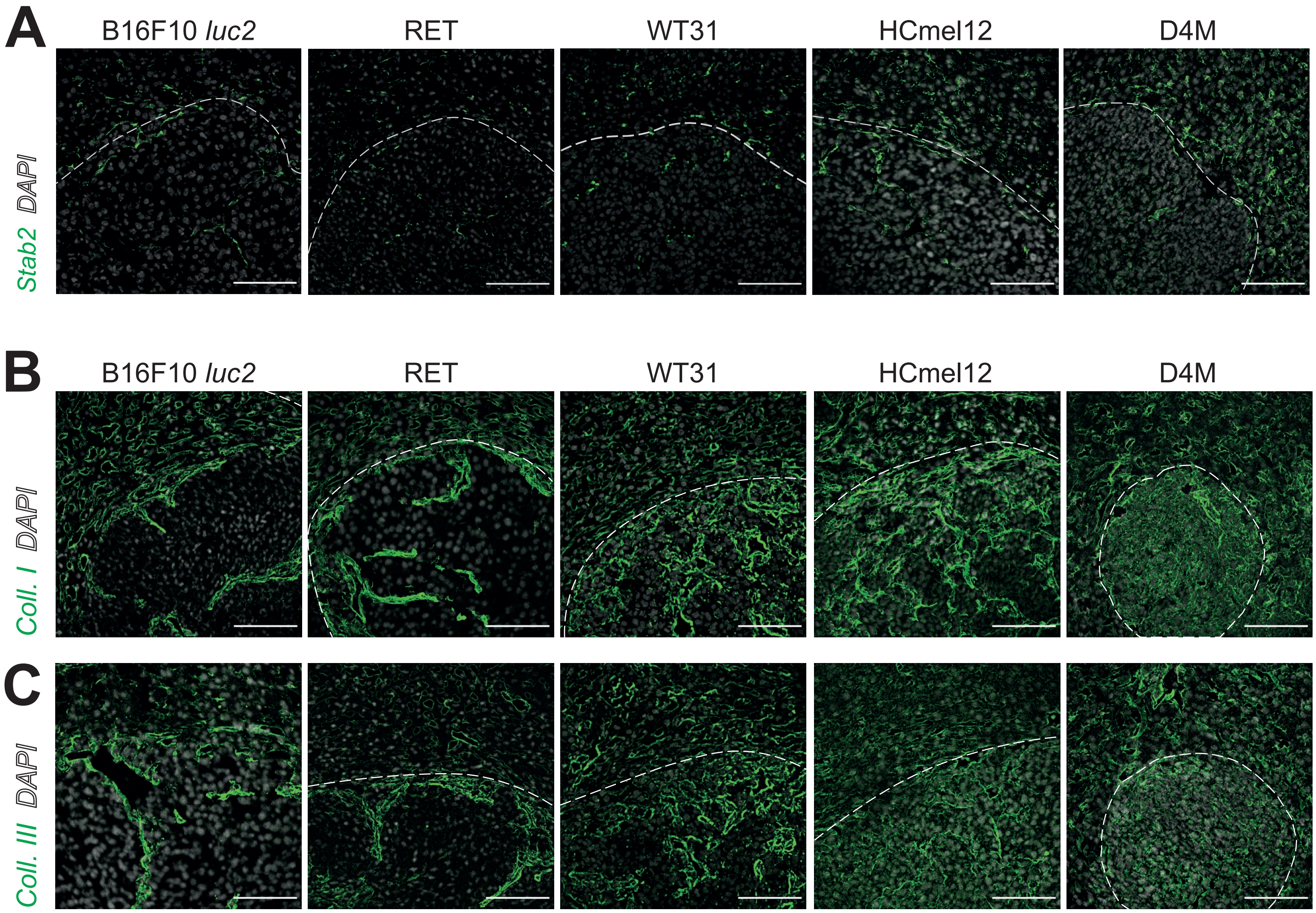

Suppl. Figure 8

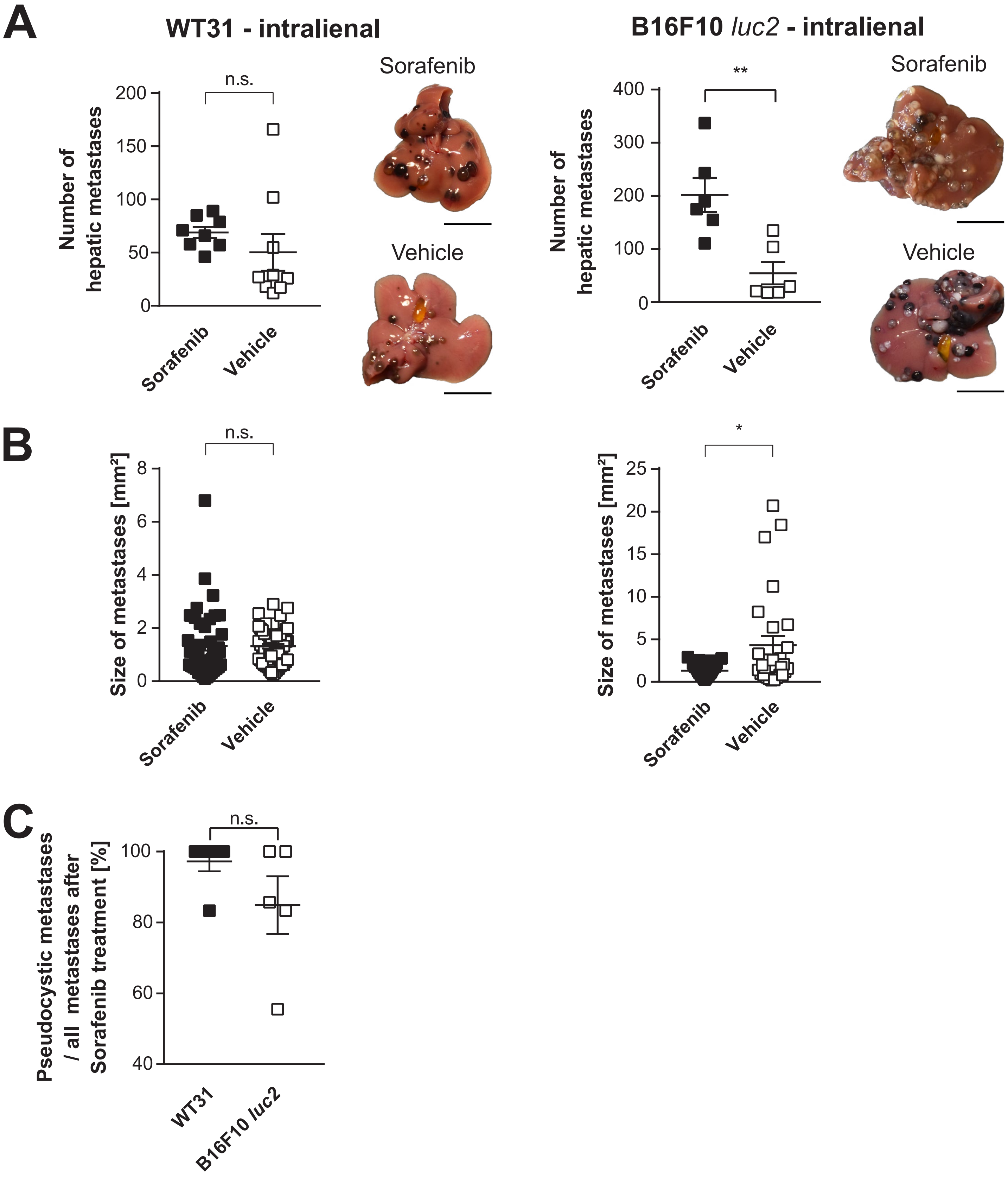

Supplement: Supplementary file 1 — Additional file 1: Figure S1. Morphological characterization of melanoma cell lines. A. A selection of representative pictures of hepatic metastases of WT31, B16F10 luc2, RET, D4M and HCmel12 melanoma after spleen injection. Besides, liver metastases of WT31 melanoma after i.v. injection are shown. Scale bar = 1 cm. B. Images of cell pellets of B16F10 luc2, RET, WT31, HCmel12 and D4M melanoma. C. Representative pictures of lungs, brain, tibia, kidneys and spleen with metastases after intravenous injection of WT31 melanoma. Scale bar = 1 cm. Figure S2. Sirius red and PAS stainings of hepatic metastases of B16F10 luc2, RET, WT31, HCmel12 and D4M. A. Images of Sirius red (SR) stainings of hepatic metastases of B16F10 luc2, RET, WT31, HCmel12 and D4M melanoma. Scale bar = 200 µm, n ≥ 5. B. Pictures of liver metastases of B16F10 luc2, RET, WT31, HCmel12 and D4M melanoma stained by PAS. Scale bar = 200 µm, n ≥ 5. Figure S3. Heat map of top25 up- and downregulated genes and heat map of melanocytic differentiation genes. A. A heatmap of the 25 most up- and downregulated genes of HIM-melanoma in comparison to LM-melanoma is shown. Please also refer to Fig. 3A, B for definition of this gene set. B. A heat map of common melanocytic differentiation genes is presented. A red color code presents an upregulation of genes, whereas the green color code shows downregulation. Clustering of genes is presented on the left side. Figure S4. Heat map GOBP—cell migration. A heat map of the GOBP cell migration gene set is presented for B16F10, RET, WT31, HCmel12 and D4M melanoma cells. The heat map was split in two parts to fit the page. A red color code presents an upregulation of genes, whereas the green color code shows downregulation. Clustering of genes is presented on the left side. Figure S5. Heat map GOBP—angiogenesis. A heat map of the GOBP angiogenesis gene set is presented for B16F10, RET, WT31, HCmel12 and D4M melanoma cells. A red color code presents an upregulation of genes, wh [file 12967_2022_3255_MOESM1_ESM.pdf]
