# Supplementary material for: Angiogenic and molecular diversity determine hepatic melanoma metastasis and response to anti-angiogenic treatment
Source: J Transl Med. 2022 Feb 2;20:62. doi: 10.1186/s12967-022-03255-4 (PMC8812268; doi:10.1186/s12967-022-03255-4)
Supplement: Supplementary file 2 — Additional file 2: Table S1. Statistic analysis of metastatic efficiency and number of hepatic metastases of B16F10 luc2, RET, WT31, HCmel12 and D4M after spleen injection. Table S2. A. Percentage of animals with hepatic metastasis after spleen injections of melanoma cells. B. Number of hepatic metastases of each individual animal after spleen injections of melanoma cells. Table S3. A. Percentage of animals with hepatic metastasis after intravenous injections of WT31 melanoma cells. B. Number of hepatic metastases of each individual animal after intravenous injections of WT31 melanoma cells. Table S4. Sequences of primers used for qPCRs. Table S5. Statistic analysis of qPCRs of Kazn, St6galnac3, Glrb, Tyr, Unc5c, Thbs2, Tfap2b, Tenm3, Stambpl1 and Ly6e in WT31, B16F10 luc2, RET, D4M and HCmel12 melanoma cells. [file 12967_2022_3255_MOESM2_ESM.docx]

**Additional tables**

**Table S1.** Statistic analysis of metastatic efficiency and number of hepatic metastases of B16F10 *luc2*, RET, WT31, HCmel12 and D4M after spleen injection

| Pairwise Comparison | Efficiency of hepatic colonization (Fig. 1A) | Number of hepatic metastases (Fig. 1B) |
| --- | --- | --- |
| B16F10 – RET | 0.0345 | 0.0028 |
| B16F10 – WT31 | 0.3113 | 0.1133 |
| B16F10 – Hcmel12 | 0.0006 | 0.0011 |
| B16F10 – D4M | < 0.0001 | < 0.0001 |
| RET – WT31 | 0.0275 | 0.0016 |
| RET – Hcmel12 | 0.1447 | 0.4000 |
| RET – D4M | 0.0036 | 0.0060 |
| WT31 – Hcmel12 | 0.0025 | 0.0011 |
| WT31 – D4M | < 0.0001 | < 0.0001 |
| Hcmel12 – D4M | 0.2404 | 0.1290 |

**A.** Pairwise comparisons of the efficiency of hepatic colonization (Fig. 1A) or number of hepatic metastasis are presented (Fig. 1B). Mann-Whitney U-tests were performed.

**Table S2. A.** Percentage of animals with hepatic metastasis after spleen injections of melanoma cells

| **Number of cells injected** | **Melanoma cell line** | **Hepatic metastasis** | | | | | | | | | |
| --- | --- | --- | --- | --- | --- | --- | --- | --- | --- | --- | --- |
| 10000 | WT31 | 1 | 1 | 1 |  |  |  |  |  |  |  |
| 30000 | WT31 | 1 | 1 | 1 |  |  |  |  |  |  |  |
| 50000 | B16F10 *luc2* | 1 | 0 | 1 | 0 | 1 | 0 |  |  |  |  |
| 100000 | B16F10 *luc2* | 1 | 1 | 1 | 1 |  |  |  |  |  |  |
| 150000 | B16F10 *luc2* | 1 | 1 | 0 | 0 | 0 | 1 | 1 | 1 | 1 | 1 |
| 50000 | RET | 1 | 0 | 1 | 0 | 1 | 0 | 0 |  |  |  |
| 100000 | RET | 1 | 1 | 0 | 0 | 0 | 0 |  |  |  |  |
| 150000 | RET | 1 | 1 | 1 | 0 | 1 | 1 | 1 | 0 | 0 | 1 |
| 50000 | D4M | 0 | 0 | 0 | 0 | 0 |  |  |  |  |  |
| 100000 | D4M | 0 | 0 | 0 | 0 | 0 |  |  |  |  |  |
| 150000 | D4M | 0 | 0 | 0 | 0 | 0 |  |  |  |  |  |
| 300000 | D4M | 0 | 0 | 0 | 0 | 1 | 0 | 1 |  |  |  |
| 50000 | HCmel12 | 0 | 0 | 0 | 0 | 0 |  |  |  |  |  |
| 100000 | HCmel12 | 0 | 0 | 0 | 0 | 0 |  |  |  |  |  |
| 150000 | HCmel12 | 0 | 0 | 0 | 0 | 0 |  |  |  |  |  |
| 300000 | HCmel12 | 1 | 1 | 1 | 1 | 0 | 1 | 1 |  |  |  |

**A.** Animals with (1) or without (0) hepatic metastases after spleen injection of melanoma cells with a certain number of cells are shown.

**Table S2 B.** Number of hepatic metastases of each individual animal after spleen injections of melanoma cells

| **Number of cells injected** | **Melanoma cell line** | **Number of hepatic metastases** | | | | | | | | | |
| --- | --- | --- | --- | --- | --- | --- | --- | --- | --- | --- | --- |
| 10000 | WT31 | 16 | 4 | 1 |  |  |  |  |  |  |  |
| 30000 | WT31 | 250 | 59 | 64 |  |  |  |  |  |  |  |
| 50000 | B16F10 *luc2* | 3 | 0 | 16 | 0 | 10 | 0 |  |  |  |  |
| 100000 | B16F10 *luc2* | 2 | 5 | 10 | 1 |  |  |  |  |  |  |
| 150000 | B16F10 *luc2* | 12 | 9 | 0 | 0 | 0 | 8 | 4 | 29 | 38 | 1 |
| 50000 | RET | 2 | 0 | 2 | 0 | 1 | 0 | 0 |  |  |  |
| 100000 | RET | 1 | 4 | 0 | 0 | 0 | 0 |  |  |  |  |
| 150000 | RET | 40 | 11 | 2 | 0 | 1 | 1 | 12 | 0 | 0 | 1 |
| 50000 | D4M | 0 | 0 | 0 | 0 | 0 |  |  |  |  |  |
| 100000 | D4M | 0 | 0 | 0 | 0 | 0 |  |  |  |  |  |
| 150000 | D4M | 0 | 0 | 0 | 0 | 0 |  |  |  |  |  |
| 300000 | D4M | 0 | 0 | 0 | 0 | 1 | 0 | 403 |  |  |  |
| 50000 | HCmel12 | 0 | 0 | 0 | 0 | 0 |  |  |  |  |  |
| 100000 | HCmel12 | 0 | 0 | 0 | 0 | 0 |  |  |  |  |  |
| 150000 | HCmel12 | 0 | 0 | 0 | 0 | 0 |  |  |  |  |  |
| 300000 | HCmel12 | 3 | 19 | 27 | 25 | 0 | 5 | 3 |  |  |  |

**B.** The numbers of hepatic metastases per animal after spleen injection of melanoma cells with a certain number of cells are shown.

**Table S3. A.** Percentage of animals with hepatic metastasis after intravenous injections of WT31 melanoma cells

| **Number of cells injected** | **Melanoma cell line** | **Hepatic metastasis** | | | | | | | | | | | | | | | |
| --- | --- | --- | --- | --- | --- | --- | --- | --- | --- | --- | --- | --- | --- | --- | --- | --- | --- |
| 1,25 x 10^6 | WT31 | 1 | 1 | 1 | 0 | 1 | 1 |  |  |  |  |  |  |  |  |  |  |
| 1,75 x 10^6 | WT31 | 1 | 1 | 1 | 1 | 1 |  |  |  |  |  |  |  |  |  |  |  |
| 2,5 x 10^6 | WT31 | 1 | 1 | 1 | 1 | 1 | 1 | 1 | 1 | 1 | 1 | 1 | 1 | 1 | 1 | 1 | 1 |

**A.** Animals with (1) or without (0) hepatic metastases after spleen injection of melanoma cells with a certain number of cells are shown.

**Table S3 B.** Number of hepatic metastases of each individual animal after intravenous injections of WT31 melanoma cells

| **Number of cells injected** | **Melanoma cell line** | **Number of hepatic metastases** | | | | | | | | | | | | | | | |
| --- | --- | --- | --- | --- | --- | --- | --- | --- | --- | --- | --- | --- | --- | --- | --- | --- | --- |
| 1,25 x 10^6 | WT31 | 51 | 28 | 6 | 0 | 1 | 59 |  |  |  |  |  |  |  |  |  |  |
| 1,75 x 10^6 | WT31 | 1 | 8 | 2 | 1 |  |  |  |  |  |  |  |  |  |  |  |  |
| 2,5 x 10^6 | WT31 | 5 | 2 | 2 | 3 | 8 | 5 | 19 | 28 | 24 | 11 | 22 | 10 | 20 | 10 | 30 | 41 |

**B.** The numbers of hepatic metastases per animal after intravenous injection of WT31 melanoma cells with a certain number of cells are shown.

**Table S4.** Sequences of primers used for qPCRs

| **Gene** | **Seq. forward** | **Seq. reverse** |
| --- | --- | --- |
| Kazn | GCGATATCGACCTGAAGGAGTA | TGAGTATGTGCTTCCCGCTG |
| St6galnac3 | GACTGGAAAGGACAGAGTCCA | GTAGCAGGCATCCATAGCCA |
| Glrb | ATCAGCACTTTGCAGGTTGGT | GTTCTTTGCCTGTGGTTTCCC |
| Tyr | AACACACTGGAAGGATTTGCC | GTTGGCCGATCCCTGTACTT |
| Unc5c | ACTGTACTGTGTCAGAGGAACC | TAGCTTCTGCCGGATAGGGA |
| Thbs2 | AGACAGGCTACATGAGAGTCTTAG | ACCAGCGTAGGTTTGGTCAT |
| Tfap2b | ACCTCACTGGTAGAAGGGGAA | TTGGTGGCCAGCAGCATATT |
| Tenm3 | TGGTGACGTTTGGCTTCCAT | TCCAAAGATGGGCGGCAC |
| Stambpl1 | TCCCCAAAGCATAAAGACACCG | TGAACAGCTTGGGGTCCTTG |
| Ly6e | TGCGGGCTTTGGGAATGT | TTGGCAGCAGTAGCTGTTCA |

**Table S5.** Statistic analysis of qPCRs of Kazn, St6galnac3, Glrb, Tyr, Unc5c, Thbs2, Tfap2b, Tenm3, Stambpl1 and Ly6e in WT31, B16F10 *luc2*, RET, D4M and HCmel12 melanoma cells

| **Gene** | **Cell line** | **compared to cell line** | **p-Value** |
| --- | --- | --- | --- |
| Kazn | Wt31 | D4M | <,0001* |
|  | Wt31 | HCmel12 | <,0001* |
|  | Wt31 | RET | <,0001* |
|  | B16F10 luc2 | D4M | 0,0003* |
|  | B16F10 luc2 | HCmel12 | 0,0003* |
|  | B16F10 luc2 | RET | 0,0004* |
|  | Wt31 | B16F10 luc2 | 0,2096 |
|  | RET | D4M | 0,9999 |
|  | RET | HCmel12 | 0,9999 |
|  | HCmel12 | D4M | 1 |
| St6galnac3 | B16F10 luc2 | HCmel12 | <,0001* |
|  | B16F10 luc2 | D4M | <,0001* |
|  | Wt31 | HCmel12 | <,0001* |
|  | Wt31 | D4M | <,0001* |
|  | B16F10 luc2 | RET | <,0001* |
|  | RET | HCmel12 | <,0001* |
|  | RET | D4M | <,0001* |
|  | B16F10 luc2 | Wt31 | <,0001* |
|  | Wt31 | RET | 0,0002* |
|  | D4M | HCmel12 | 1 |
| Glrb | Wt31 | HCmel12 | <,0001* |
|  | Wt31 | D4M | <,0001* |
|  | B16F10 luc2 | HCmel12 | <,0001* |
|  | B16F10 luc2 | D4M | <,0001* |
|  | Wt31 | RET | <,0001* |
|  | RET | HCmel12 | <,0001* |
|  | RET | D4M | <,0001* |
|  | B16F10 luc2 | RET | <,0001* |
|  | Wt31 | B16F10 luc2 | 0,0009* |
|  | D4M | HCmel12 | 0,9998 |
| Tyr | Wt31 | D4M | <,0001* |
|  | Wt31 | HCmel12 | <,0001* |
|  | Wt31 | RET | <,0001* |
|  | Wt31 | B16F10 luc2 | <,0001* |
|  | B16F10 luc2 | D4M | <,0001* |
|  | B16F10 luc2 | HCmel12 | <,0001* |
|  | B16F10 luc2 | RET | <,0001* |
|  | RET | D4M | 0,1137 |
|  | RET | HCmel12 | 0,142 |
|  | HCmel12 | D4M | 0,9999 |
| Unc5c | RET | D4M | <,0001* |
|  | RET | HCmel12 | <,0001* |
|  | B16F10 luc2 | D4M | <,0001* |
|  | B16F10 luc2 | HCmel12 | <,0001* |
|  | Wt31 | D4M | <,0001* |
|  | Wt31 | HCmel12 | <,0001* |
|  | RET | Wt31 | <,0001* |
|  | B16F10 luc2 | Wt31 | <,0001* |
|  | RET | B16F10 luc2 | 0,9917 |
|  | HCmel12 | D4M | 0,9999 |
| Ly6e | D4M | Wt31 | <,0001* |
|  | D4M | B16F10 luc2 | <,0001* |
|  | D4M | RET | <,0001* |
|  | D4M | HCmel12 | <,0001* |
|  | HCmel12 | Wt31 | 0,3288 |
|  | HCmel12 | B16F10 luc2 | 0,3288 |
|  | HCmel12 | RET | 0,3296 |
|  | RET | Wt31 | 1 |
|  | RET | B16F10 luc2 | 1 |
|  | B16F10 luc2 | Wt31 | 1 |
| Stambpl1 | D4M | Wt31 | <,0001* |
|  | D4M | B16F10 luc2 | <,0001* |
|  | D4M | RET | <,0001* |
|  | HCmel12 | Wt31 | <,0001* |
|  | HCmel12 | B16F10 luc2 | <,0001* |
|  | HCmel12 | RET | <,0001* |
|  | D4M | HCmel12 | 0,0048* |
|  | RET | Wt31 | 1 |
|  | RET | B16F10 luc2 | 1 |
|  | B16F10 luc2 | Wt31 | 1 |
| Tenm3 | D4M | B16F10 luc2 | <,0001* |
|  | D4M | RET | <,0001* |
|  | D4M | Wt31 | <,0001* |
|  | D4M | HCmel12 | <,0001* |
|  | HCmel12 | B16F10 luc2 | 0,2011 |
|  | HCmel12 | RET | 0,2011 |
|  | HCmel12 | Wt31 | 0,2544 |
|  | Wt31 | B16F10 luc2 | 0,9999 |
|  | Wt31 | RET | 0,9999 |
|  | RET | B16F10 luc2 | 1 |
| Tfap2b | D4M | B16F10 luc2 | <,0001* |
|  | D4M | Wt31 | <,0001* |
|  | D4M | RET | <,0001* |
|  | HCmel12 | B16F10 luc2 | <,0001* |
|  | HCmel12 | Wt31 | <,0001* |
|  | HCmel12 | RET | <,0001* |
|  | D4M | HCmel12 | 0,0289* |
|  | RET | B16F10 luc2 | 1 |
|  | Wt31 | B16F10 luc2 | 1 |
|  | RET | Wt31 | 1 |
| Thbs2 | D4M | B16F10 luc2 | <,0001* |
|  | D4M | Wt31 | <,0001* |
|  | D4M | RET | <,0001* |
|  | HCmel12 | B16F10 luc2 | <,0001* |
|  | HCmel12 | Wt31 | <,0001* |
|  | HCmel12 | RET | <,0001* |
|  | D4M | HCmel12 | 0,0289* |
|  | RET | B16F10 luc2 | 1 |
|  | Wt31 | B16F10 luc2 | 1 |
|  | RET | Wt31 | 1 |
